# Brown Carbon from Photo-Oxidation of Glyoxal and SO_2_ in Aqueous Aerosol

**DOI:** 10.1021/acsearthspacechem.3c00035

**Published:** 2023-04-27

**Authors:** David O. De Haan, Lelia N. Hawkins, Praveen D. Wickremasinghe, Alyssa D. Andretta, Juliette R. Dignum, Audrey C. De Haan, Hannah G. Welsh, Elyse A. Pennington, Tianqu Cui, Jason D. Surratt, Mathieu Cazaunau, Edouard Pangui, Jean-François Doussin

**Affiliations:** † Department of Chemistry and Biochemistry, University of San Diego, 5998 Alcala Park, San Diego, California 92117, United States; ‡ Department of Chemistry, Harvey Mudd College, 301 Platt Blvd, Claremont, California 91711, United States; § Department of Environmental Sciences and Engineering, Gillings School of Global Public Health, University of North Carolina at Chapel Hill, Chapel Hill, North Carolina 27599, United States; ∥ Department of Chemistry, College of Arts and Sciences, University of North Carolina at Chapel Hill, Chapel Hill, North Carolina 27599, United States; ⊥ Laboratoire Interuniversitaire des Systèmes Atmosphériques (LISA), UMR7583, CNRS, Institut Pierre Simon Laplace (IPSL), Université Paris-Est-Créteil (UPEC) et Université Paris Diderot (UPD), Créteil 94010, France

**Keywords:** photoreduction, oligomer, redox, sulfate formation, photosensitizer, photobrowning, photobleaching, viscosity

## Abstract

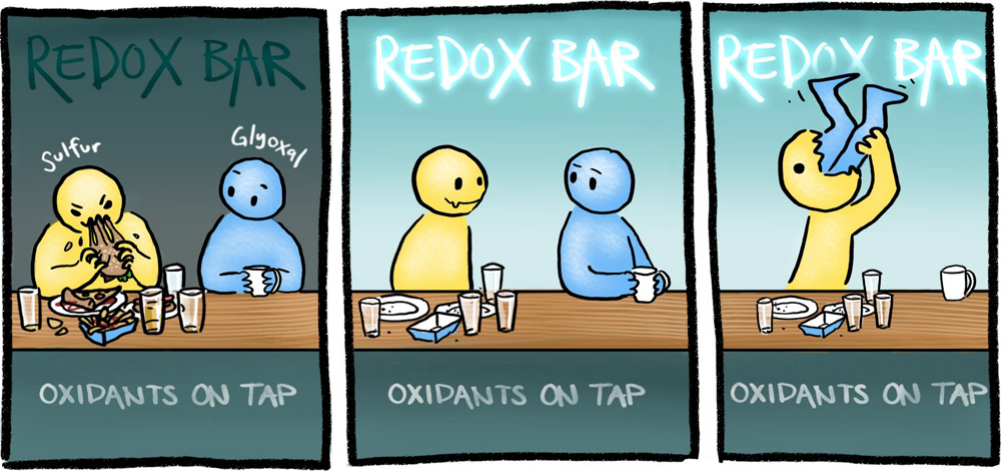

Aqueous-phase dark reactions during the co-oxidation of glyoxal and S(IV) were recently identified as a potential source of brown carbon (BrC). Here, we explore the effects of sunlight and oxidants on aqueous solutions of glyoxal and S(IV), and on aqueous aerosol exposed to glyoxal and SO_2_. We find that BrC is able to form in sunlit, bulk-phase, sulfite-containing solutions, albeit more slowly than in the dark. In more atmospherically relevant chamber experiments where suspended aqueous aerosol particles are exposed to gas-phase glyoxal and SO_2_, the formation of detectable amounts of BrC requires an OH radical source and occurs most rapidly after a cloud event. From these observations we infer that this photobrowning is caused by radical-initiated reactions as evaporation concentrates aqueous-phase reactants and aerosol viscosity increases. Positive-mode electrospray ionization mass spectrometric analysis of aerosol-phase products reveals a large number of C_*x*_H_*y*_O_*z*_ oligomers that are reduced rather than oxidized (relative to glyoxal), with the degree of reduction increasing in the presence of OH radicals. This again suggests a radical-initiated redox mechanism where photolytically produced aqueous radical species trigger S(IV)–O_2_ auto-oxidation chain reactions, and glyoxal-S(IV) redox reactions especially if aerosol-phase O_2_ is depleted. This process may contribute to daytime BrC production and aqueous-phase sulfur oxidation in the atmosphere. The BrC produced, however, is about an order of magnitude less light-absorbing than wood smoke BrC at 365 nm.

## Introduction

Glyoxal and S(IV) react through aqueous-phase reactions to reversibly form the sulfonate adduct molecules glyoxal monobisulfite and glyoxal di-bisulfite (GDBS).^1^ Sulfonate adducts are resistant to oxidation by ozone and H_2_O_2_^2^ and are thus more stable than their precursor species. As a result, they are important reservoir species in the atmosphere, increasing the partitioning of both glyoxal and SO_2_ to the aqueous phase.^3^ Recently, it was shown that reactions in 1:1 mixtures of glyoxal and bisulfite ions (dissolved SO_2_) in the presence of trace oxidants from the air can rapidly form oligomerized brown carbon (BrC) species, along with C_1_ and C_3_ sulfonate products, under slightly acidic conditions.^4^ The C_1_ sulfonate product, hydroxymethylsulfonate, has been detected at high concentrations in aerosol,^5−7^ but was previously assumed to be formed only by the formaldehyde + S(IV) reaction.^7−9^

Secondary BrC formation in clouds and aqueous aerosol can potentially worsen climate change by absorbing solar radiation, a process known as the direct aerosol effect. The direct radiative forcing caused by the BrC component of aerosol has been estimated at 0.13 ± 0.01 W m^–2^.^10−13^ In areas often impacted by biomass burning (e.g., the tropical mid and upper troposphere), BrC radiative forcing is greater than that of black carbon,^14^ although generally it is less.^15,16^ All in all, BrC impacts are difficult to assess because its aging processes are not well understood.^17^

Many BrC mixtures have been shown via bulk aqueous experiments to be very susceptible to photobleaching^18−23^ and oxidation by ozone,^24,25^ which has led to estimates of atmospheric BrC lifetimes that vary between ∼30 min for BrC products derived from aldehyde + ammonium sulfate (AS) reactions^20,26,27^ to several hours^18,21,28−31^ to more than a day,^22,25^ depending on the BrC source and aging process. In contrast, recent studies where mixtures of amines, AS, and carbonyl species were irradiated in the aqueous aerosol phase reported photobrowning lasting a few hours.^32,33^ Other lab and field studies have observed initial browning of BrC mixtures followed by bleaching after several hours of reaction time,^26,28,29,31,34^ or even more complicated time-dependent changes.^35^ This diversity of results across BrC aging studies indicates that there is no reason to expect that BrC produced by different source reactions will behave similarly. Furthermore, the photobleaching and photobrowning behavior of glyoxal + S(IV) reactions has not been measured.

Here, we report measurements of the negative effects of sunlight on BrC formation in aqueous bulk mixtures of glyoxal and S(IV) at pH 5.5. In suspended aqueous aerosol particles exposed to glyoxal and SO_2_ and undergoing cloud processing in a large chamber, we find that BrC formation is observed only in the presence of OH radical-initiated oxidation (using the photolysis of H_2_O_2_ as the OH radical source). Oligomerized aqueous-phase reaction products with unexpectedly low carbon oxidation states are explored via (+)-mode electrospray ionization mass spectrometry.

## Methods

All solutions were made in 18 MΩ deionized water from solid reagents supplied by Sigma-Aldrich, unless otherwise specified. No unexpected or unusually high safety hazards were encountered.

### Bulk Aqueous Experiments

To test the effects of sunlight on glyoxal + S(IV) mixtures, duplicate samples were created containing glyoxal (hydrolyzed from a solid trimer by stirring in deionized water overnight, Fluka, >99%), pH 5.5 acetate buffer, and sodium sulfite (Na_2_SO_3_, Spectrum) solution. This slightly acidic pH was selected because it is near the middle of the pH range observed for cloud droplets and sea spray aerosol.^36^ Sample pairs in glass vials (with transmittance 50% cutoffs at ∼350 nm) were placed in direct afternoon sunlight (late September, clear sky, 32°46′N, noon to 4 pm) in 4 h intervals, with sunlight blocked to one of each pair by aluminum foil, such that temperatures for sunlit and shaded samples remained within 2 °C of each other, with shaded samples typically at the higher temperature. During each 4 h reaction interval, solar insolation declined from ∼1.0 to 0.8 kW/m^2^. The UV/vis absorbance spectrum of each sample was measured in 1 cm pathlength quartz cuvettes after each 4 h reaction interval, and the sample was stored overnight at 4 °C until the next sunny afternoon’s solar irradiation interval. Precipitates formed upon cooling certain reaction samples were identified by powder X-ray diffraction (XRD, Bruker Apex II DUO) by comparison to authentic standards.

### CESAM Chamber Experiments

To quantify the effects of simulated sunlight, OH radicals, and multiphase chemistry on glyoxal + S(IV) chemistry, aerosol seed particles were generated from 9 mM sodium sulfate (>99%) solution using an atomizer (TSI 3076) in experiments performed in the presence of SO_2_ gas (Merck, >99.95%). In experiments without SO_2_ gas, particulate S(IV) was introduced by atomizing 9 mM sodium sulfite (Fluka, >99%) solution. Since 9 mM Na_2_SO_3_ solutions are slightly basic (pH ∼7.5), these solutions were buffered in most experiments to pH 5.5 using sulfuric acid. Gas-phase glyoxal was generated from a heated mixture of solid glyoxal trimer dihydrate and solid P_2_O_5_ (>99%),^37^ and introduced into the chamber in either a pulse (filling a glass bulb on a vacuum line to a well-determined pressure and flushing the contents into the chamber) or continuously (flowing dry N_2_ through the solid mixture at ∼140 °C into the chamber). Resulting glyoxal concentrations ranged from 150 to 640 ppb and were quantified by proton transfer reaction–mass spectrometry (PTR–MS) (KORE II) after calibration of signals by long-path in situ Fourier transform infrared (FTIR) spectroscopy using standard spectra.^38^ During segments of certain experiments, hydrogen peroxide was added as an OH precursor by bubbling a 2 L/min flow of O_2_ through a bubbler containing 30% HOOH (ACS-grade, nonstabilized) into the chamber.

CESAM is a pressure and temperature controlled stainless steel smog/cloud chamber with a fixed volume of 4.2 m^3^ and three 6500 W Xe solar simulator lamps. Sampling flows are automatically compensated by additions of N_2_ (evaporated from liquid) and high-purity O_2_ at an 80:20 ratio. Gas-phase contents were monitored by an SO_2_ sensor, long-path FTIR (Bruker Tensor), PTR–MS, and sensors for temperature, RH, ozone, and NO_x_. Dried aerosol properties, including total optical scattering/extinction and size distributions, were measured by cavity-attenuated phase shift/single-scattering albedo spectroscopy (CAPS-ssa, Aerodyne, 450 nm) and a scanning mobility particle sizer (SMPS, TSI 3080/3772), respectively. Aerosol and cloud droplet size distributions were also characterized by light scattering (PALAS welas Digital 2000, 0.4–15 μm range). At the end of each experiment, chamber-processed aerosol samples were collected on Teflon filters (1.0 μm pore size, 47 mm diam., Tisch Sci.) and frozen at −20 C until extraction and off-line ultrahigh-performance liquid chromatography coupled with (+)-mode electrospray ionization high-resolution quadrupole time-of-flight mass spectrometry (UHPLC/ESI-HR-QTOFMS) analysis. Aerosol optical properties were also monitored without drying by particle into liquid sampler (PILS) sampling into a waveguide UV/vis spectrometer with a 1 m pathlength and an inline total organic carbon (TOC) monitor. Gas-phase signals were corrected for dilution from N_2_ additions, and SMPS signals were corrected for dilution and wall losses. CAPS-ssa signals were corrected using second-order polynomial fits to daily calibrations with dried AS aerosol. Waveguide UV/vis data were normalized to TOC levels to generate mass absorption coefficients (MACs) in cm^2^/gOC using the equation MAC = 2.303*A*/*bC*, where *A* is the measured log_10_ absorbance at a given wavelength, *b* is the pathlength in cm, and *C* is the TOC level in g organic carbon cm^–3^. The clean chamber was used for the waveguide reference spectrum. Due to temperature-dependent variations in detector response and lamp output, baselines often drifted to slightly negative absorbance values. During periods with measurable chromophores, the absorbance spectra appeared as expected. However, due to this drift, true MAC values may be slightly larger than our reported values.

All Teflon filter samples collected from chamber experiments were extracted with methanol (Optima LC/MS Grade, Fisher Scientific) by sonication for 45 min. The methanol extracts were dried under a gentle stream of high-purity nitrogen gas (Airgas) and reconstituted in 150 μL of 50:50 methanol and Milli-Q water. Filter extracts were analyzed by UHPLC/ESI-HR-QTOFMS operated in positive (+) ion mode, as previously described in detail.^39^ Aliquots of 5–10 μL were injected onto a Waters ACQUITY UPLC HSS T3 column (2.1 × 100 mm, 1.8 μm particle size) and eluted at 0.3 mL min^–1^ with methanol and water solvent mixtures containing 0.1% ammonium acetate (LC/MS Chromasolv-grade, Sigma-Aldrich). Data were analyzed using Agilent MassHunter Version B.06.00 Build 6.0.633.0 qualitative software.

## Results and Discussion

### Bulk Aqueous Experiments

During solar irradiation, glyoxal/sulfite (HSO_3_^–^ at pH 5.5) samples had average temperatures of 35.5 and 37.5 °C with and without exposure to direct sunlight, respectively. After four hours of exposure and overnight refrigeration, a white precipitate formed in glyoxal/HSO_3_^–^ samples, with higher quantities in samples that were shielded from solar radiation. This precipitate was identified as GDBS by comparing its powder XRD spectra with an authentic GDBS standard. GDBS is known to be formed reversibly in mixtures of glyoxal and sulfite, and is considered a reservoir species^1^ in oxidant-free solution.^4^

**Figure 1:**
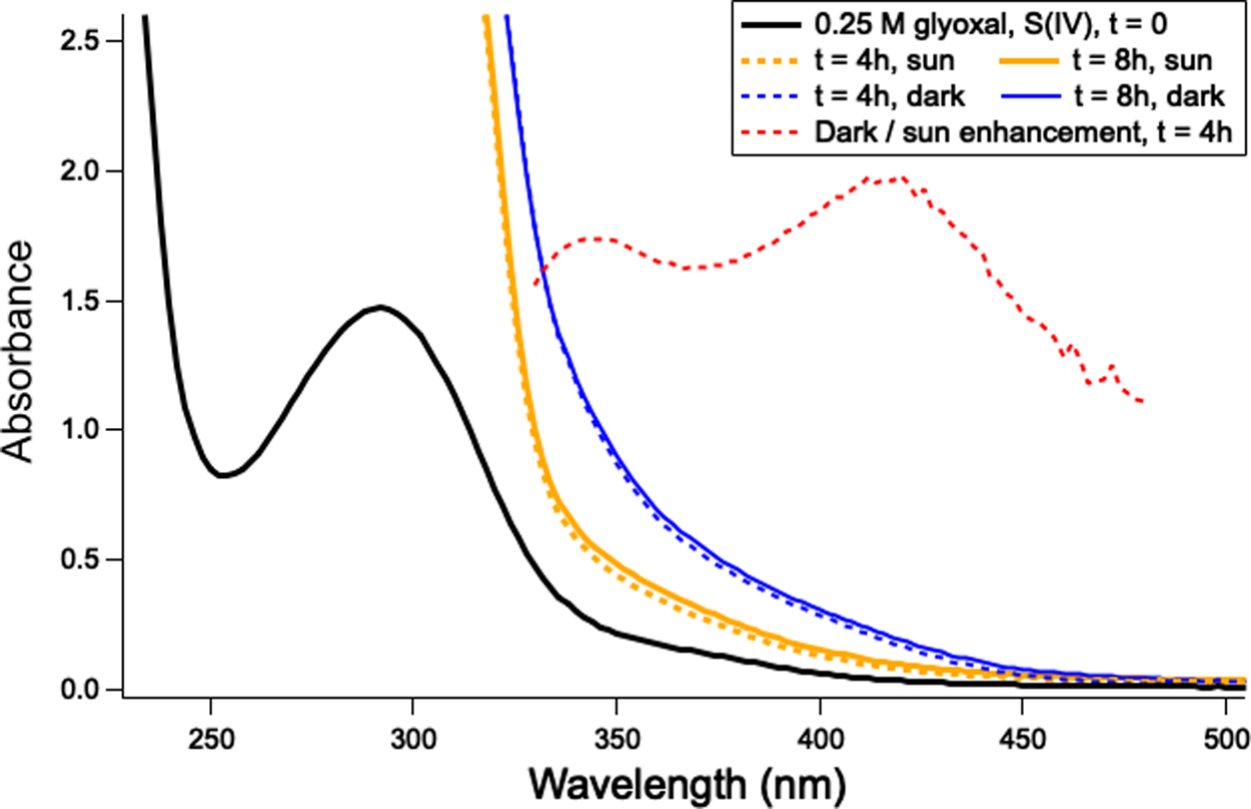
Absorption spectrum of filtered pH 5.5 reaction mixture containing 0.25 M glyoxal and 0.25 M HSO_3_^–^ before (black line) and after one and two 4 h periods of solar irradiation (gold) or dark processing at similar temperatures (blue). 4 h reaction time (dashed lines); 8 h reaction time (solid colored lines). Ratio of dark versus sun absorption enhancement (dashed red line) is shown for wavelengths beyond 320 nm; the average ratio between 330 and 480 is 1.6 ± 0.2.

Absorbance spectra of filtered 1:1 glyoxal:HSO_3_^–^ mixtures are shown in Figure 1. Modest absorbance in the actinic range was observed initially upon sample mixing, along with an absorbance maximum at 292 nm observed in an earlier study.^4^ After 4 h of sunlight exposure, absorbance was saturated below 330 nm and enhanced by factors of 2.0–3.4 out to 480 nm. (Absorbance was below detection limits beyond 480 nm.) In duplicate temperature-matched samples shielded from sunlight, the absorbance was enhanced by factors between 3.3 and 5.5 over the same wavelength range, a change which is much larger than run-to-run variation (∼15%). The enhancement in absorbance in the dark was larger than in sunlight at all wavelengths between 330 and 480 nm, with an average dark/sunlight enhancement ratio of 1.6 ± 0.2. These results demonstrate that photobleaching of BrC products formed by glyoxal + sulfite reactions occurs, but this photobleaching cannot keep pace with BrC production in these sunlit (250 mM) solutions where no OH precursor was added.

### CESAM Chamber Experiments with Gas-Phase Glyoxal and SO_2_

Glyoxal + S(IV) experiments conducted in the CESAM chamber are summarized in Table 1. Glyoxal concentrations (150–640 ppb) were chosen to match an earlier study of glyoxal + S(IV) dark chemistry. In experiments 1–4, 140–600 ppb SO_2_ gas was added to the chamber, and dried seed aerosol particles were generated from Na_2_SO_4_ solutions. The SO_2_ concentration range was chosen to simulate an extremely polluted atmosphere (bracketing China’s 1 h “grade II” air quality standard for SO_2_ of 500 ppb), and to approach the predicted SO_2_ gas-phase concentration if gas-aqueous equilibrium were reached in experiments 5–7 (670 ppb), where dried seed aerosol particles were produced from Na_2_SO_3_ solution as the S(IV) source. The sulfite solution was buffered to pH 5.5 with sulfuric acid in Experiments 6 and 7, resulting in aerosol initially consisting of mixed, effloresced NaHSO_3_ and NaHSO_4_. The pH of the unbuffered Na_2_SO_3_ solution used to generate seed aerosol particles in Experiment 5 was ∼7.5.

**Table 1:** CESAM Chamber Cloud Processing Experiments with S(IV) and Gas-Phase Glyoxal^a^

expt.	figure	[GX]_g_(ppb)^b^	seed aerosol types^c^	[SO_2_]_g_ (ppb)	[HOOH]_g_added	secondary aerosol produced^d^ (μg/m^3^)	MAC max (cm^2^/gOC) 365 nm	min. albedo reached, 450 nm	Δ albedo (final cloud)
1	Figure 2	170 ± 10	Na_2_SO_4_	140 ± 5	yes, cloud 3	57 ± 4	100 ± 40 (no data after cloud 3)	0.75 ± 0.02	–0.15 ± 0.03
2	Figure S3	170 then 310	Na_2_SO_4_	550	yes, precloud only	40	380	0.83	–0.03
3	Figure S4	640	Na_2_SO_4_^e^	520	no	26	120	0.86	–0.012
4	Figure 3	150	Na_2_SO_4_^e^	600	yes, cloud 2	65	1800	0.89	–0.06
5	Figure S2	170	Na_2_SO_3_	8 ± 1^f^	yes, cloud 3	20	2000	0.92	noisy
6	Figure S1	150	pH 5.5 Na_2_SO_3_	10 ± 1^f^	no	6	2600	0.88	noisy
7	Figure S7	490	pH 5.5 Na_2_SO_3_ in N_2_	20 ± 1^f^	no	6	1200	0.85	–0.02 w/RH

a Notes: all runs had 2–3 cloud events, with solar simulator lights turned on for the last cloud event (except in Experiment 2, where lights were on under dry conditions only followed by two dark cloud events). Uncertainties listed for Experiment 1 are typical for all experiments unless otherwise stated.

b Based on PTR–MS signals at *m*/*z* 31 and on pressure in glass transfer bulb in Experiment 1, calibrated by long-path FTIR.

c Diffusion dried and suspended in high-purity air unless otherwise stated.

d Measured by SMPS, assuming an aerosol density of 1.0.

e Flash-dried (liquid particles sent into dry chamber).

f Equilibrated from sulfite seed particles, concentration given is that measured after seed particle addition was complete. Higher levels were briefly observed upon humidification, see the corresponding figures.

**Figure 2:**
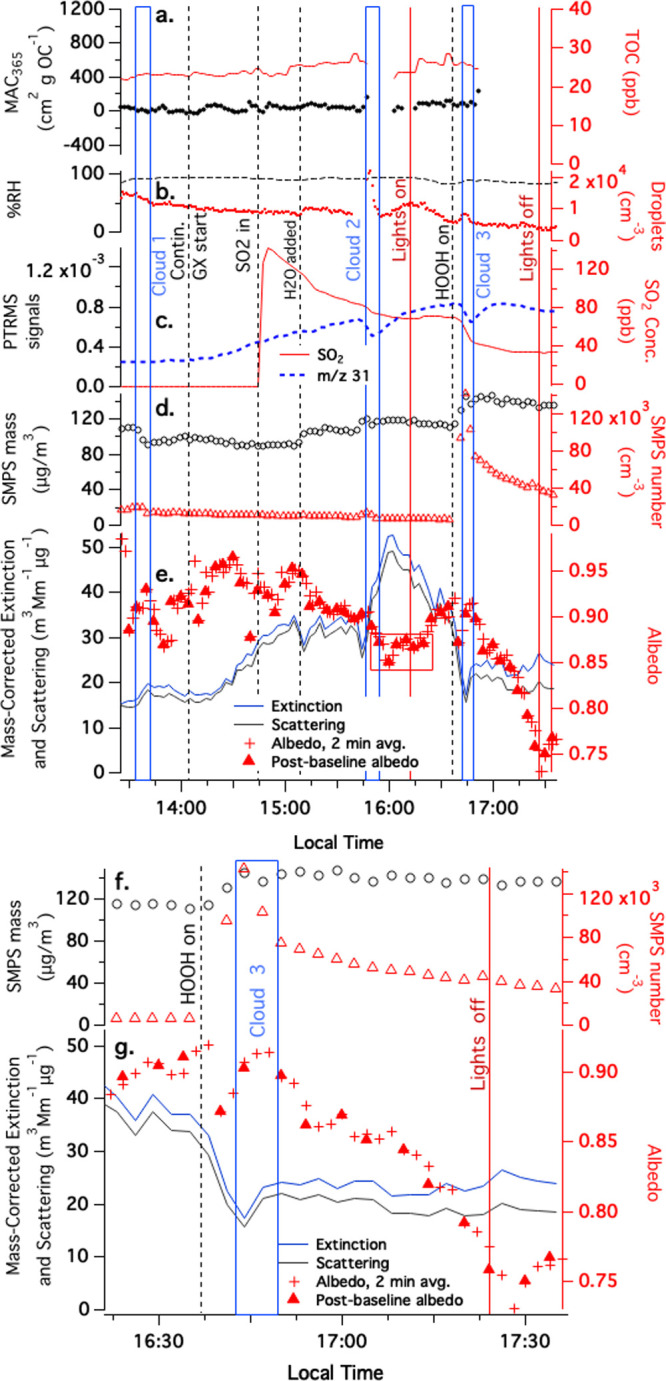
Gas-phase glyoxal and SO_2_ uptake during Experiment 1 with deliquesced Na_2_SO_4_ aerosol in CESAM chamber, 9:50 start time. Three cloud events, start of continuous glyoxal addition, SO_2_ addition, start and end of chamber illumination, and start of HOOH addition are labeled. Panel a: TOC readings and MACs at 365 nm from PILS/waveguide UV–vis, (re-zeroed at 16:03), color-coded to axes. (b) Chamber RH and droplet spectrometer counts, color-coded to axes. (c) Water- and dilution-corrected PTR–MS data (*m*/*z* 31 glyoxal fragment, blue dotted line); and SO_2_ concentrations in ppb from a dedicated sensor (red line). (d) SMPS total mass (assuming density = 1 g/cm^3^) and counts shown next, color-coded to axes. (e) CAPS-ssa data at 450 nm (mass-corrected extinction, blue line; mass-corrected scattering, black line; single-scattering albedo, red dots; ssa measured immediately after gas-phase baseline, red triangles; red box indicates points measured beyond calibration range of instrument). (f and g) Copies of panels d and e, zoomed in on end of experiment to better show onset times of nucleation event and browning.

In Experiment 1 (Figure 2), gas-phase glyoxal and SO_2_ were added to the chamber containing deliquesced sodium sulfate aerosol at RH > 90%. Glyoxal was added continuously after 14:03 (local time), while SO_2_ was added in a single pulse at 14:44. Neither of these additions caused a significant increase in dried particle mass or browning, although TOC rose gradually by 10% starting after the SO_2_ addition, likely due to some aqueous-phase glyoxal-SO_2_ adduct formation.

Normally, chamber cloud events cause a decrease in dried particle mass that is measurable by SMPS, due to wet deposition of some cloud droplets containing the aerosol particles that served as cloud condensation nuclei. (The lifetime of a 200 nm diameter particle in the chamber is on the order of a day, but when activated into a 5 μm cloud droplet, its lifetime shortens to several minutes.) An example of this wet deposition can be seen in the 18% dried particle mass loss after cloud 1 at 13:33, or 8–15% mass losses after the clouds in Figures S1 and S2. In contrast, once glyoxal and SO_2_ gases were present in the chamber, a water vapor addition at 15:15 and cloud 2 caused respective increases in particle mass of 22 and 12%. The fact that TOC signals do not also increase in a correlated manner with SMPS mass indicates that this increase in particle mass is mainly due to sulfite or sulfate formation. Since the Henry’s law coefficient of SO_2_ is only 1.47 M/atm, dissolved SO_2_ (or H_2_SO_3_) concentrations are expected to remain below 1 μM, unless a base is present to react with H_2_SO_3_ to produce HSO_3_^–^ or SO_3_^2–^ ions. Indeed, negligible aerosol-phase sulfur was observed in an earlier study where wet NaCl aerosol was exposed to SO_2_(g).^4^ A proton transfer reaction involving sulfate ions is thus likely responsible for the dark growth in aerosol dry mass observed in Experiment 1:
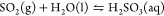

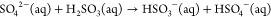


After the chamber solar simulator lights were turned on, HOOH(g) was added to the chamber as an OH radical source, about a minute before cloud event 3. As soon as HOOH was added, particle counts increased more than 10-fold and particle mass increased by 27% due to a nucleation event, likely caused by oxidation of SO_2_ by HOOH. (The identity of the oxidant as HOOH and not OH radicals is supported by Experiment 2 (Figure S3), where new particle nucleation is seen in the dark when SO_2_(g) and HOOH(g) are present and RH reaches 25%.) During cloud event 3, glyoxal (g) concentrations temporarily fell from 120 to 90 ppb while SO_2_(g) concentrations fell from 71 to 43 ppb. While the temporary loss of glyoxal was similar to that observed during the previous (HOOH-free and dark) cloud event 2, the drop in SO_2_(g) concentrations during cloud 3 was several times larger than during cloud 2, indicating the importance of SO_2_ + HOOH reactions in driving SO_2_ uptake to the aqueous phase.

No detectable BrC formed during the dark portion of Experiment 1: no rise in MAC at 365 nm or convincing drop in albedo at 450 nm was observed. As cloud 3 dissipated, however, the single-scattering albedo measured at 450 nm began to decline from 0.90 to 0.75, stopping only when the lights were turned off 40 min later. Therefore, this photobrowning was driven by either direct photolysis or by OH radical reactions, and occurred most rapidly after the evaporation of cloud droplets. Furthermore, it appears that neither cloud events nor photolysis/OH radical-initiated photo-oxidation are sufficient to cause uptake and rapid photobrowning by glyoxal and SO_2_ in aqueous aerosol particles; rather, both are required.

**Figure 3:**
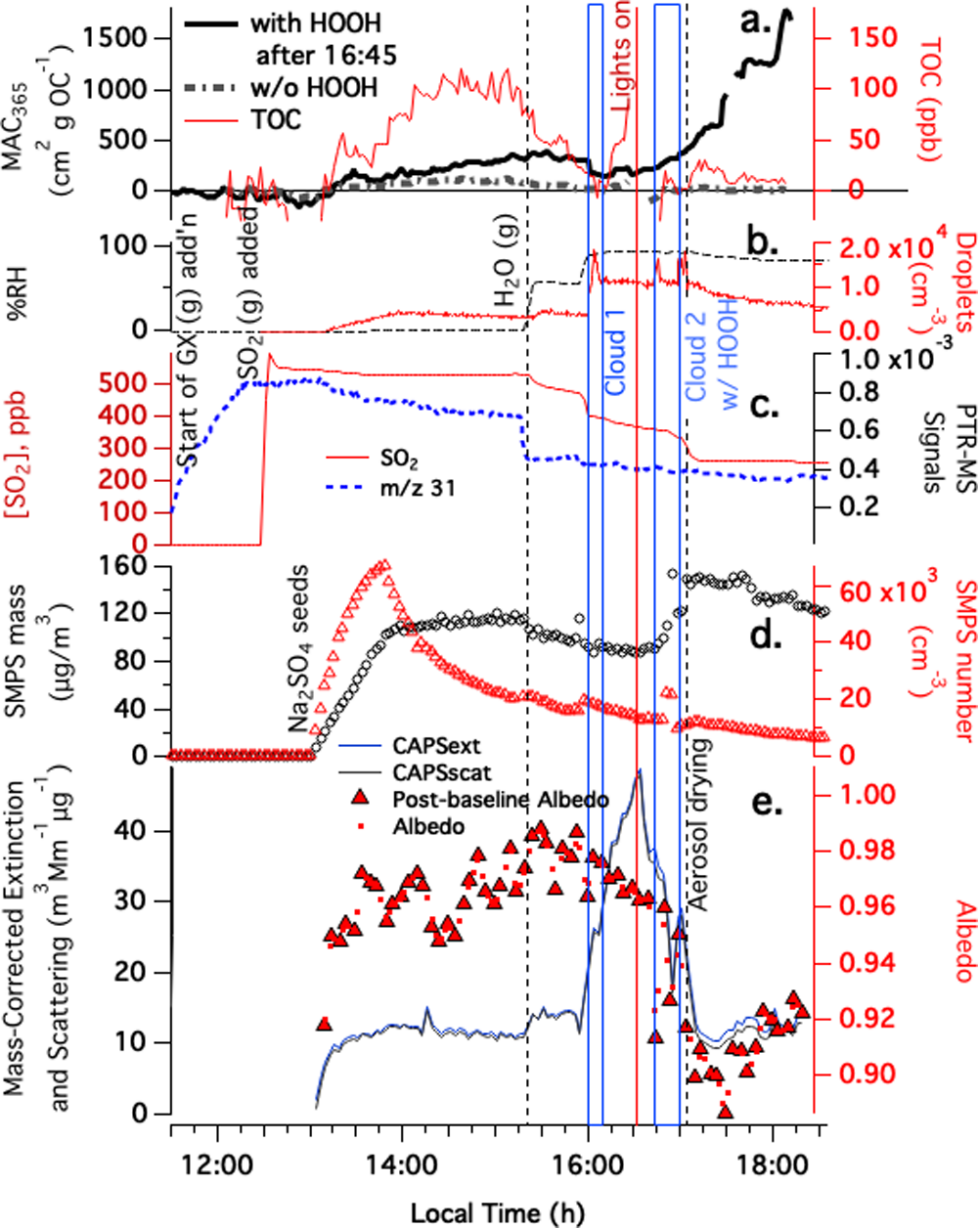
Gas-phase glyoxal and SO_2_ uptake Experiment 4 with Na_2_SO_4_ aerosol in the CESAM chamber, 8:31 start time. Glyoxal gas added continuously after 11:30. SO_2_ addition, water vapor addition, two cloud events, start of chamber illumination by solar simulator lights, and the onset of aerosol drying on the way to SMPS and CAPS-ssa instruments are labeled. Panel a: MACs measured by PILS—waveguide—TOC at 365 nm in Expt. 4 with HOOH during cloud 2 (red line) compared with Expt. 3 (no HOOH added, gray dash-dot line, complete data in Figure S4). (b) chamber RH and droplet spectrometer counts, color-coded to axes. (c) Water- and dilution-corrected PTR–MS data from chamber (*m*/*z* 31 glyoxal fragment, blue dotted line) and SO_2_ concentrations in ppb from a dedicated sensor (red line). (d) SMPS total mass (assuming density = 1 g/cm^3^) and counts, color-coded to axes. (e) CAPS-ssa data at 450 nm (mass-corrected extinction, blue line; mass-corrected scattering, black line; 2 min averaged single-scattering albedo, red dots, and albedo measured immediately after instrument baselines, red triangles).

Similar experiments on BrC formation from SO_2_(g) and GX(g) uptake onto Na_2_SO_4_ seeds, but with (Experiment 4) and without HOOH addition (Experiment 3) during the final cloud event, are compared in Figure 3. In both experiments, cloud 1 occurred in the dark without HOOH, and did not produce secondary organic aerosol (SOA) mass or BrC, as before. Albedo at 450 nm declined by less than 0.012, and the MAC at 365 nm increased by less than 80 cm^2^ g^–1^, both within the noise of these data sets. In Experiment 3, cloud 2 occurred with the lights on but in the absence of HOOH. Under these conditions, no BrC, and less than 5 μg/m^3^ of aerosol, was produced (Figure S4). In contrast, in Experiment 4 with lights and HOOH, cloud 2 produced 65 μg/m^3^ of secondary aerosol and substantial BrC, visible from the large increase in MAC and large decrease in albedo in Figure 3. As before, a drop in albedo at 450 nm and an increase in MAC at 365 nm occur once light, HOOH, and a cloud event are all present, suggesting that aqueous photobrowning reactions involving OH radicals are responsible for BrC production. (The very small MAC increase at 365 nm before cloud 2 is likely due to limited photobrowning involving lower levels of radical generation by direct photolysis or photosensitization.)

### CESAM Chamber Experiments with Sulfite Aerosol

In a previous study, BrC production was observed when gas-phase glyoxal interacted with aqueous sulfite-containing aerosol particles in a flowing system with a 1 min residence time, without the need for OH radical sources or sunlight.^4^ Experiments 5–7 (Figures S1, S2, S5, and S6) explore this system to try to better understand differences between experiments where S(IV) is initially supplied in the gas or aerosol phase. In all three experiments, dried sulfite-containing aerosol were added to the dry chamber, accompanied by relatively small amounts (8–20 ppb) of gas-phase SO_2_, likely released from the aqueous phase during initial aerosol generation (before drying). The addition of glyoxal gas under dry conditions (RH < 5%) did not increase SMPS mass or MAC at 365 nm. This is consistent with previous flow chamber experiments with sulfite-containing aerosol, where glyoxal did not cause aerosol browning or substantial growth under dry conditions.^4^

In Experiments 5–7, once the chamber was humidified, most glyoxal gas was immediately lost from the gas phase, and TOC levels increased, showing that glyoxal was taken up by the deliquesced, sulfite-containing aerosol particles. At the same time, SO_2_ gas was released from the aerosol to the gas phase as equilibrium with aqueous-phase sulfite was established. However, most of the SO_2_ gas released upon aerosol deliquescence was recaptured within 15 min, presumably due to aqueous reactions with glyoxal occurring in aerosol particles and on chamber walls. In Experiment 5, SO_2_ recapture resulted in net growth of the aerosol, while in experiments where the aerosol was acidified to pH 5.5 (Experiments 6 and 7), no net growth was observed. In all three experiments, a large increase in MAC was observed upon SO_2_ recapture under dark conditions, consistent with the dark production of BrC previously observed for this system.^4^ These observations are strong evidence that glyoxal can form light-absorbing BrC in dark reactions with high concentrations of bisulfite ions in deliquesced, mildly acidic aerosol, but not under dry conditions.

Since aerosol deliquescence is enough to cause glyoxal uptake and browning with Na_2_SO_3_ or NaHSO_3_ aerosol, even in the dark and without HOOH, it appears that the mechanism of BrC formation is different when starting with gas-phase SO_2_ as compared to aerosol-phase sulfite. In bulk solution, or when deliquesced, sulfite-containing aerosol is exposed to gas-phase glyoxal, and glyoxal-sulfite adduct molecules evidently reach such high concentrations in the aqueous phase that BrC can form in the dark, as observed in both types of experiments. However, when gas-phase SO_2_ is the S(IV) source, glyoxal-sulfite adduct molecules produced after the uptake of both SO_2_ and glyoxal to the aqueous phase are likely present at much lower concentrations. Under these conditions, which are more relevant to the atmosphere, the formation of detectable amounts of BrC oligomers requires an OH radical source and occurs most rapidly after a cloud event.

### Mass Spectral Analysis of Chamber-Processed Aerosol

**Figure 4:**
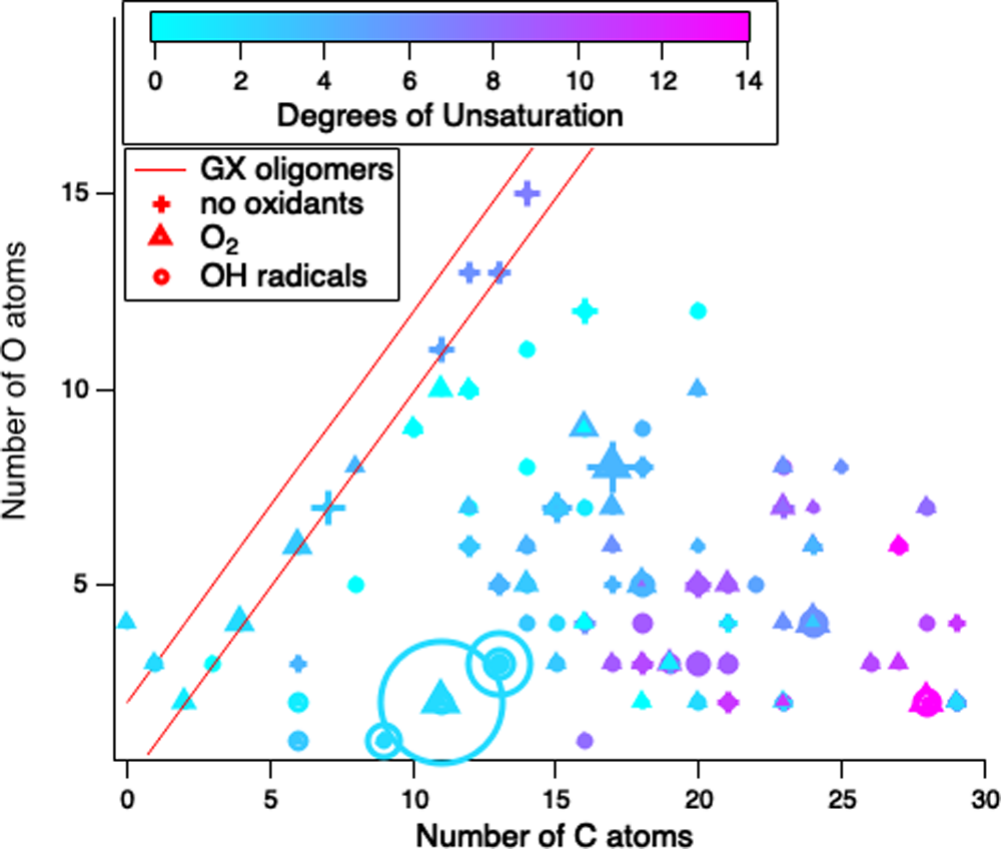
Summary of molecular formula detected by UHPLC/(+)ESI-HR-QTOFMS analysis of filter extracts of chamber aerosol in Experiments 1 and 3–7, graphed in terms of numbers of O and C atoms. Symbols indicate oxidant present: no oxidant (+), and O_2_ (triangles), or OH radicals (circles). Colors indicate degrees of unsaturation, symbol areas are proportional to average peak areas across experiments with the given oxidant, and red lines indicate expected range of particulate glyoxal oligomers. Multiple symbols of the same shape and color centered on one location represent isomers with identical mass but distinguished by retention times.

The molecular formulas of compounds detected by UHPLC/(+)ESI-HR-QTOFMS in filter extracts from Experiments 1 and 3–7 are shown in Figures 4 and S7 as well as in Table S1. The molecules detected in the aerosol phase depended most strongly on the oxidant present in the chamber, rather than on the source of S(IV). In Experiment 7, performed without oxidants (the aerosol was suspended in N_2_ containing no more than a few ppm O_2_, and no HOOH was added at any point), a much higher fraction of detected organic aerosol molecules fell within the O/C ratio range expected for glyoxal oligomers, shown in Figure 4 as the region between the two red lines. These oligomers include C_7_–C_14_ species, which we note are larger than the C_4_ dimers and C_6_ trimers commonly detected in aqueous GX mixtures. These larger particulate-phase oligomers likely formed during the evaporative stage of cloud processing.

The ion-count-weighted average parameters of molecules detected by UHPLC/(+)ESI-HR-QTOFMS in each experiment are summarized as a function of oxidant used in Table 2. In the absence of O_2_ and HOOH but with sunlight, the average oxidation state of carbon in detected product molecules was −0.45, significantly lower than carbon’s +1.0 oxidation state in glyoxal and its oligomers. This suggests that significant aerosol-phase redox reactions have taken place, with S(IV) species presumably serving as reducing agents. Since glyoxal and S(IV) are known to form stable adduct molecules through aqueous phase reactions when oxidants are excluded,^1^ the aerosol redox reactivity we observed may be triggered by radical species formed by direct photolysis of BrC species or by photosensitization. These radicals could initiate direct redox chemistry between S(IV) and glyoxal, allowing the formation of reduced organic species along with sulfate ions.

**Table 2:** Average Parameters of Molecules Detected by UHPLC/(+)ESI-HR-QTOFMS in Aerosol Particles as a Function of Oxidant Used in Glyoxal + S(IV) + Sunlight-Experiments^a^

average parameter	no oxidant	O_2_		OH radical		
experiment number	7	3	6	1	4	5
degrees of unsaturation	5.0	5.4	4.8	6.0	3.2	5.8
		*x̅* = 5.1 ± 0.4		*x̅* = 4.0 ± 1.6		
O/C ratio	0.52	0.42	0.40	0.39	0.24	0.34
		*x̅* = 0.41 ± 0.01		*x̅* = 0.28 ± 0.08		
C atoms/molecule	16.5	17.0	16.6	17.9	13.7	18.2
		*x̅* = 16.8 ± 0.3		*x̅* = 15.1 ± 2.5		
C oxidation state	–0.45	–0.68	–0.79	–0.72	–1.24	–0.79
		*x̅* = −0.73 ± 0.07		*x̅* = −1.09 ± 0.3		

a Notes: average values for each experiment are weighted by MS ion counts for each detected species. Averages (with uncertainties) calculated from multiple experiments are shown as *x̅* values.

In Experiments 3 and 6, performed in N_2_/O_2_ mixtures instead of only N_2_ gas, average carbon oxidation state and average O/C ratio in detected products both declined further. In the presence of O_2_, S(IV) auto-oxidation is known to be initiated by radical species or transition metals, and this auto-oxidation involves a catalytic chain reaction where sulfoxy radicals are intermediates.^40^ In our experiments, any contribution to S(IV) auto-oxidation from trace metal contaminants is likely suppressed by glyoxal,^41^ so we can assume that photolytically produced radical species or BrC photosensitization starts the S(IV) auto-oxidation chain reaction. Sulfoxy radicals, like OH radicals, can react with organic species by abstracting hydrogen atoms^42^ or adding to C=C double bonds.^43^ Addition of sulfoxy radicals to C=C double bonds would generate organosulfate species, which do not ionize well in (+)-mode ESI due to their negative charge.^44^ Indeed, only 3 out of the 126 aqueous aerosol species detected by (+)-mode ESI-MS contained sulfur in our experiments, indicating that only the H-abstraction reaction pathway is being probed here. Since organic species with more double bonds have fewer hydrogens and higher carbon oxidation states and are more susceptible to radical addition than species with fewer double bonds, our use of (+)-mode electrospray ionization likely exhibits a detection bias toward more reduced products. However, similar values for average degree of unsaturation across all types of experiments, with their different radical levels, suggests that this bias is rather small. Indeed, Walser et al.^45^ compared positive and negative mode ESI-MS analysis of SOA produced by limonene ozonolysis and found that the (+)-mode ESI bias in the measured average O/C was only −0.07 (O/C = 0.43 in (+)-mode versus 0.50 in (−)-mode, both significantly higher than O/C = 0 in the reactant limonene). In the current work, the O/C ratio difference between the reactants (glyoxal and its oligomers with O/C ≥ 1) and the products detected by (+)-mode ESI are 7–10× larger than this detection bias.

In the presence of dissolved O_2_ neither hydrogen abstraction nor radical addition would be expected to form products with lower O/C ratios than the reactants, since O_2_ normally adds to organic radical species produced by either pathway. Thus, the additional decline in both carbon oxidation state and O/C ratios in experiments where O_2_ was present suggests that aerosol-phase O_2_ is depleted by the time oligomers form. In other words, after launching the aqueous-phase auto-oxidation of S(IV) and the production of sulfoxy radicals, resulting in greater production of organic radicals, O_2_ may become depleted in evaporating aqueous aerosol due to molecular crowding (increased sinks) and increased viscosity, which slows O_2_ diffusion into the particles. If aerosol-phase O_2_ is depleted, organic radicals can react more readily with other nearby organic species, producing oligomers with lower O/C ratios, as observed. O_2_ depletion in aerosol particles, or the related change in photochemical products to photoreduction and oligomerization, has been observed in recent studies of viscous aerosol phases.^23,46^ Additionally, aqueous aerosol containing glyoxal has been documented to become highly viscous.^47^ We hypothesize that the evaporation of cloud droplets in the atmosphere can create similarly viscous aerosol particles, which then have the potential to become depleted in O_2_.

With the addition of HOOH as an OH radical precursor, the measured average O/C ratios and carbon oxidation states of aerosol phase organic species drop even further than that caused by the addition of O_2_. This result, while again counter-intuitive, is the same trend as before. Positive-mode ESI-MS also has a detection bias against organic acids, which are the products of OH radical addition in the presence of dissolved O_2_, but this detection bias is again unlikely to explain simultaneous declines in O/C ratios and carbon oxidation states in the presence of OH radicals. Instead, the declines are likely due to higher initial radical concentrations triggering more S(IV) auto-oxidation chain reactions, resulting in more sulfoxy radicals producing greater numbers of organic radical species, which more quickly deplete aqueous-phase O_2_ and then form less-oxygenated oligomers. We note, however, that the average number of carbon atoms per detected molecule does not change significantly under the different oxidant conditions. This suggests that different oxidant conditions are changing the type of oligomers formed in the glyoxal + S(IV) system (more oxidized or more reduced), rather than causing large changes in the quantity or total extent of oligomerization.

Finally, we note that in experiments where OH radicals were present, the average degree of unsaturation in detected organic aerosol species does not change significantly from other experiments. Since the formation of BrC species requires the production of molecules with delocalized π bonds in order to absorb visible light,^17,48^ one might expect the average degree of unsaturation to increase in this system, where BrC was formed. Instead, these results indicate that the majority of the detected organic aerosol species are not light-absorbing BrC molecules. Instead, a small minority of highly absorbing species are apparently responsible for the optical properties of BrC aerosol formed by reactions between S(IV) and glyoxal, as has been observed in other reaction systems that form BrC.^49^

### Atmospheric Significance

These laboratory experiments show that gas-phase glyoxal and SO_2_ can be taken up by cloud droplets and, in the presence of an OH radical source, will form sulfate and reduced (rather than oxidized) oligomerized species including BrC. From these observations, we infer that radical-initiated redox reactions between SO_2_ and glyoxal have taken place in O_2_-depleted, postcloud aqueous aerosol particles. The role of glyoxal in these reactions is likely twofold. First, like any small aldehyde, glyoxal reacts with dissolved SO_2_ to form sulfonate adducts,^1,3,8,50,51^ which keep the two reactant species in physical proximity and, we hypothesize, make subsequent redox reactions between them more likely. Second, glyoxal oligomerization increases the viscosity of aqueous aerosol,^47,52^ making O_2_ depletion more likely. These roles suggest that other combinations of (1) small, water-soluble, sulfonate-adduct-forming aldehydes, plus (2) oligomer-forming organic molecules might also be able to generate the conditions necessary for radical-initiated SO_2_—aldehyde redox reactions and BrC oligomer production in the aqueous aerosol phase.

While the concentrations of glyoxal (>150 ppb) and SO_2_ (>140 ppb) used in these studies were very high, the total aldehyde concentration in Beijing can approach ∼100 ppb,^53^ and SO_2_ concentrations as high as 50 ppb were measured in 2010 in the lower planetary boundary layer over eastern China.^54^ Furthermore, estimates of global aerosol phase have shown that most aerosol particles are semisolids, especially in the middle and upper troposphere.^55^ Thus, it may be possible that SO_2_ can engage in redox reactions with small aldehyde molecules in semisolid aerosol particles in many regions of the troposphere.

The BrC MAC_365_ levels of 0.12–0.26 m^2^ gOC^–1^ (or 1200–2600 cm^2^ gOC^–1^) measured in experiments 4–7 compare with BrC MAC_365_ mean levels of 0.8–2.4 m^2^ gOC^–1^ recently measured in Asian cities^56,57^ and with 0.5–5 m^2^ gOC^–1^ BrC MAC_365_ measured in wood smoke.^58,59^ The secondary BrC produced by glyoxal + SO_2_ reactions in this work therefore appears to be approximately an order of magnitude less absorbing than BrC from wood smoke.

## Data Availability

Concentration–time profiles for the large chamber experiments are freely accessible in .edf format through the chamber database at data.eurochamp.org maintained by AERIS for the benefit of ACTRIS ERIC. (Expt. 1: https://doi.org/10.25326/4HS3-M215. Expt 2: https://doi.org/10.25326/XE7Z-EC10. Expt. 3: https://doi.org/10.25326/5X59-Q090. Expt. 4: https://doi.org/10.25326/D3DD-Y688. Expt 5: https://doi.org/10.25326/J1ZN-X623. Expt 6: https://doi.org/10.25326/R7T9-8X80. Expt 7: https://doi.org/10.25326/4841-QM92.)

## Supporting Information Available

Aerosol-phase compounds detected in each experiment by UHPLC/ESI-HR-QTOFMS, summary plots for chamber experiments 2, 3, and 5–7, a sample of time-dependent UV–vis spectra in experiment 7 from water-soluble aerosol particles collected by PILS, and graph summarizing the O/C ratio, H/C ratio, and degree of unsaturation of detected aerosol-phase compounds (PDF)
