# Correction to Brown Carbon from Photo-Oxidation of Glyoxal and SO_2_ in Aqueous Aerosol

**DOI:** 10.1021/acsearthspacechem.3c00144

**Published:** 2023-06-05

**Authors:** David O. De Haan, Lelia N. Hawkins, Praveen D. Wickremasinghe, Alyssa D. Andretta, Juliette R. Dignum, Audrey C. De Haan, Hannah G. Welsh, Elyse A. Pennington, Tianqu Cui, Jason D. Surratt, Mathieu Cazaunau, Edouard Pangui, Jean-François Doussin

**Affiliations:** † Department of Chemistry and Biochemistry, University of San Diego, 5998 Alcala Park, San Diego, California 92117, United States; ‡ Department of Chemistry, Harvey Mudd College, 301 Platt Blvd, Claremont, California 91711, United States; § Department of Environmental Sciences and Engineering, Gillings School of Global Public Health, University of North Carolina at Chapel Hill, Chapel Hill, North Carolina 27599, United States; ∥ Department of Chemistry, College of Arts and Sciences, University of North Carolina at Chapel Hill, Chapel Hill, North Carolina 27599, United States; ⊥ Université Paris-Est Créteil and Université Paris Cité, CNRS, LISA, F-94010 Créteil, France

The affiliation listed for authors Mathieu Cazaunau, Edouard Pangui, and Jean-François Doussin has been corrected in this Addition/Correction. The correct affiliation is Université Paris-Est Créteil and Université Paris Cité, CNRS, LISA, F-94010 Créteil, France.

