# Supplementary material for: Brown Carbon from Photo-Oxidation of Glyoxal and SO2 in Aqueous Aerosol
Source: ACS Earth Space Chem. 2023 Apr 27;7(5):1131–40. doi: 10.1021/acsearthspacechem.3c00035 (PMC10201569; doi:10.1021/acsearthspacechem.3c00035)
Supplement: Supplementary file 1 — sp3c00035_si_001.pdf [file sp3c00035_si_001.pdf]

Brown Carbon from Photooxidation of Glyoxal and SO_2 in Aqueous Aerosol:

Supplemental Information

David O. De Haan,[1]* Lelia N. Hawkins,[2] Praveen D. Wickremasinghe,[1] Alyssa D. Andretta,[1] Juliette R. Dignum,[1] Audrey C. De Haan,[1] Hannah G. Welsh,[2] Elyse A. Pennington,[2] Tianqu Cui,[3,†] Jason D. Surratt,[3,4] Mathieu Cazaunau,[5] Edouard Pangui,[5] Jean-François Doussin[5]

1: Department of Chemistry and Biochemistry, University of San Diego, 5998 Alcala Park, San Diego CA 92117 USA

2: Department of Chemistry, Harvey Mudd College, 301 Platt Blvd, Claremont CA 91711 USA

3: Department of Environmental Sciences and Engineering, Gillings School of Global Public Health, University of North Carolina at Chapel Hill, Chapel Hill NC 27599 USA

4: Department of Chemistry, College of Arts and Sciences, University of North Carolina at Chapel Hill, Chapel Hill NC 27599 USA

5: Laboratoire Interuniversitaire des Systèmes Atmosphériques (LISA), UMR7583, CNRS, Université Paris-Est-Créteil (UPEC) et Université Paris Diderot (UPD), Institut Pierre Simon Laplace (IPSL), 94010 Créteil, France

[†]current address : Laboratory of Atmospheric Chemistry, Paul Scherrer Institute (PSI), 5232 Villigen-PSI, Switzerland

* Corresponding author: ddehaan@sandiego.edu, (619) 260-6882, (619) 260-2211 fax

Figure S1 – S7, Table S1.
14 pages total.

Table S1: Summary of Peaks Detected by Positive Mode ESI-MS

m/z ratio	RT (min)	Ionization	Molec formula	Δ (ppm)	degree unsat	O/C	H/C	C ox#	Expt 3 (counts)	Expt 4 (counts)	Expt 1 (counts)	Expt 5 (counts)	Expt 6 (counts)	Expt 7 (counts)
104.992702	1.27	Na^+	$NaC_2H_3O_2$	-1.3	1	1.00	1.50	0.50	2.30E+06	1.04E+05	1.34E+05		1.74E+06	1.38E+04
114.091670	7.73	NH_4^+	C_6H_8O	-1.9	3	0.17	1.33	-1.00		6.76E+06				
115.037343	1.43	Na^+	$C_3H_8O_3$	2.0	0	1.00	2.67	-0.67	2.45E+03	2.00E+06			2.66E+03	
128.953218	1.40	Na^+	Na_2CO_3	-6.5	2	3.00	0.00	6.00	1.23E+06	8.00E+04	7.13E+05	1.18E+06		
134.117615	1.52	NH_4^+	$C_6H_{12}O_2$	-3.6	1	0.33	2.00	-1.33		6.88E+06				
142.938140	1.30	Na^+	$NaHSO_4$	6.6	2				1.30E+06		8.01E+05			
149.023204	13.42	Na^+	$C_6H_6O_3$	11.7	4	0.50	1.00	0.00	7.76E+05	5.05E+05	7.47E+05	3.42E+04		8.30E+05
158.153639	6.33	NH_4^+	$C_9H_{16}O$	-5.4	2	0.11	1.78	-1.56	1.24E+04	1.89E+07	9.91E+03	5.82E+05	8.39E+05	
158.154651	6.17	NH_4^+	$C_9H_{16}O$	1.0	2	0.11	1.78	-1.56		6.51E+05		3.06E+06		
186.994553	1.31	Na^+	$Na_2C_4H_6O_4$	-7.3	2	1.00	1.50	0.50	9.32E+05	1.47E+05	2.41E+06		4.03E+06	
186.995614	1.31	Na^+	$Na_2C_4H_6O_4$	-7.3	2	1.00	1.50	0.50	5.65E+06	7.71E+05	9.03E+03	7.72E+05	6.61E+05	
195.122014	6.92	H^+	$C_8H_{19}O_5$	-6.3	0	0.63	2.25	-1.00	7.69E+04	2.05E+06		9.09E+04	1.04E+05	
202.178360	7.96	NH_4^+	$C_{11}H_{20}O_2$	-1.5	2	0.18	1.82	-1.45	5.20E+06			6.56E+06	7.04E+06	
202.180400	7.93	NH_4^+	$C_{11}H_{20}O_2$	-1.5	2	0.18	1.82	-1.45		9.33E+06				
202.183960	9.08	NH_4^+	$C_{11}H_{20}O_2$	16.0	2	0.18	1.82	-1.45		9.41E+07				
224.075043	6.82	NH_4^+	$C_7H_{10}O_7$	-8.8	3	1.00	1.43	0.57						5.44E+06
246.205625	8.76	NH_4^+	$C_{13}H_{24}O_3$	-5.3	2	0.23	1.85	-1.38	7.95E+05	1.67E+07	3.16E+05			8.34E+05
246.207166	8.79	NH_4^+	$C_{13}H_{24}O_3$	1.0	2	0.23	1.85	-1.38		4.56E+07				
255.194453	11.45	H^+	$C_{15}H_{26}O_3$	-6.1	3	0.20	1.73	-1.33	5.99E+04	3.17E+04	2.48E+06		3.11E+06	
268.997298	1.29	Na^+	$Na_3C_6H_9O_6$	-6.3	3	1.00	1.50	0.50			2.11E+06			
268.999221	1.32	Na^+	$Na_3C_6H_9O_6$	-6.3	3	1.00	1.50	0.50	6.96E+06	3.56E+05	2.25E+05			
272.179263	10.58	H^+	$C_{16}H_{21}N_3O$	10.9	8	0.06	1.31	-1.94		3.51E+06				
274.162074	9.06	NH_4^+	$C_{13}H_{20}O_5$	-12.0	4	0.38	1.54	-0.77	6.46E+04	5.84E+04	7.03E+04	1.35E+06	7.00E+04	2.06E+06
274.164786	9.01	NH_4^+	$C_{13}H_{20}O_5$	-2.4	4	0.38	1.54	-0.77			1.96E+06		1.83E+06	
278.160498	8.63	NH_4^+	$C_{12}H_{20}O_6$	0.5	3	0.50	1.67	-0.67				2.21E+06		

278.162491	8.77	NH_4^+	$C_{12}H_{20}O_6$	7.6	3	0.50	1.67	-0.67						2.12E+06
279.160159	12.91	H^+	$C_{16}H_{22}O_4$	1.9	6	0.25	1.38	-0.88	2.19E+05	5.07E+05	3.31E+05	1.79E+06	9.56E+04	2.06E+06
285.151307	11.12	H^+	$C_{18}H_{20}O_3$	7.8	9	0.17	1.11	-0.78					2.22E+06	
287.133756	11.57	H^+	$C_{10}H_{22}O_9$	-1.6	0	0.90	2.20	-0.40		3.08E+05	1.54E+06	1.43E+06		1.35E+06
287.136038	10.56	H^+	$C_{10}H_{22}O_9$	6.4	0	0.90	2.20	-0.40	1.71E+05	8.38E+05	9.02E+04	9.14E+04	1.58E+06	
287.232775	7.43	ACN-NH_4^+	$C_{13}H_{24}O_3$	-2.4	2	0.23	1.85	-1.38	4.62E+03	5.25E+06	3.88E+04	8.36E+03	3.85E+03	8.12E+03
288.160246	11.13	NH_4^+	$C_{17}H_{18}O_3$	1.0	9	0.18	1.06	-0.71	2.36E+06					
288.161791	11.06	NH_4^+	$C_{17}H_{18}O_3$	6.3	9	0.18	1.06	-0.71			2.20E+06			
295.189983	12.41	Na^+	$C_{15}H_{28}O_4$	4.9	2	0.27	1.87	-1.33	7.70E+05	2.53E+06	4.34E+05			
300.202107	7.79	NH_4^+	$C_{12}H_{26}O_7$	-0.4	0	0.58	2.17	-1.00	6.87E+04	3.89E+06	1.04E+04	4.35E+04	3.28E+04	
304.161338	10.22	NH_4^+	$C_{10}H_{22}O_9$	1.9	0	0.90	2.20	-0.40		1.51E+05	3.88E+05	9.09E+04	2.00E+06	1.18E+05
304.176426	10.28	NH_4^+	$C_{14}H_{22}O_6$	1.4	4	0.43	1.57	-0.71	1.88E+06	2.15E+05		1.96E+06	9.45E+04	1.16E+05
306.175676	10.15	NH_4^+	$C_{14}H_{24}O_4S$	5.8	3	0.29	1.71	-1.43		1.33E+06	2.10E+05	1.26E+06		
306.177745	10.14	NH_4^+	$C_{14}H_{24}O_4S$	5.8	3	0.29	1.71	-1.43					1.09E+06	1.42E+06
309.278866	13.44	Na^+	$C_{18}H_{38}O_2$	6.2	0	0.11	2.11	-1.89	1.26E+06	1.10E+06	2.52E+05		9.30E+04	5.29E+05
311.292656	12.04	H^+	$C_{20}H_{38}O_2$	-7.6	2	0.10	1.90	-1.70	3.50E+04	1.86E+06	1.43E+04			2.02E+05
311.293947	11.99	H^+	$C_{20}H_{38}O_2$	-3.4	2	0.10	1.90	-1.70	4.27E+05	1.65E+05		1.12E+06	1.24E+06	
313.235926	9.83	Na^+	$C_{16}H_{34}O_4$	1.4	0	0.25	2.13	-1.63	9.06E+05			5.23E+05	3.26E+05	3.85E+05
315.188519	9.01	ACN-NH_4^+	$C_{13}H_{20}O_5$	-11.0	4	0.38	1.54	-0.77	2.34E+04	9.10E+05	1.46E+06	1.53E+06	2.63E+04	1.09E+06
316.194604	10.73	NH_4^+	$C_{19}H_{22}O_3$	-12.0	9	0.16	1.16	-0.84	1.80E+06	5.46E+06			1.80E+06	
318.173659	10.55	NH_4^+	$C_{18}H_{20}O_4$	9.8	9	0.22	1.11	-0.67				5.89E+06	9.57E+05	4.24E+05
322.170035	10.52	NH_4^+	$C_{14}H_{24}O_5S$	3.8	3	0.36	1.71	-1.00			2.48E+06			
322.172917	10.57	NH_4^+	$C_{14}H_{24}O_5S$	12.7	3	0.36	1.71	-1.00	2.44E+06	2.20E+06			2.68E+06	
324.196835	9.37	NH_4^+	$C_{21}H_{22}O_2$	1.5	11	0.10	1.05	-0.86	8.47E+05			1.00E+06		1.21E+06
325.176846	14.47	Na^+	$C_{19}H_{26}O_3$	-3.4	7	0.16	1.37	-1.05	2.75E+06		1.27E+05			
330.204594	11.23	NH_4^+	$C_{20}H_{24}O_3$	-7.0	9	0.15	1.20	-0.90	2.21E+06	7.89E+06	3.00E+05	5.55E+05	1.83E+04	3.07E+05
330.207092	13.38	NH_4^+	$C_{20}H_{24}O_3$	0.5	9	0.15	1.20	-0.90		1.67E+06	9.71E+05	4.87E+05	1.16E+06	1.56E+04
330.227689	11.22	NH_4^+	$C_{17}H_{28}O_5$	-1.1	4	0.29	1.65	-1.06		5.94E+05	1.06E+05	1.75E+04		1.48E+06

334.167142	10.15	NH4+	C18H20O5	5.1	9	0.28	1.11	-0.56			4.68E+06	6.73E+05		
334.170745	10.30	NH4+	C11H24O10	-1.7	0	0.91	2.18	-0.36					4.56E+06	
334.186387	10.30	NH4+	C15H24O7	-0.6	4	0.47	1.60	-0.67	4.62E+06	7.03E+05	2.48E+05	4.31E+06	2.64E+05	4.85E+06
335.180731	7.98	ACN-NH4+	C12H20O7	-3.3	3	0.58	1.67	-0.50	7.26E+05			8.81E+05	8.73E+05	
336.179397	11.57	NH4+	C18H22O5	-5.1	8	0.28	1.22	-0.67	3.52E+06					
336.184001	11.46	NH4+	C18H22O5	8.6	8	0.28	1.22	-0.67		3.18E+06				
337.273969	13.14	Na+	C19H38O3	6.2	1	0.16	2.00	-1.68	9.78E+05		5.24E+05		8.51E+05	7.19E+05
340.088104	7.95	NH4+	C11H14O11	0.3	5	1.00	1.27	0.73						3.90E+06
341.184426	10.82	ACN-NH4+	C18H18O3	-6.1	10	0.17	1.00	-0.67				2.55E+06	1.45E+06	2.16E+06
344.218282	13.51	NH4+	C21H26O3	-12.4	9	0.14	1.24	-0.95	1.30E+06	4.74E+06		1.44E+06	1.59E+06	1.68E+04
344.227878	8.12	NH4+	C14H30O8	-1.6	0	0.57	2.14	-1.00		1.66E+06	1.52E+04	4.10E+05	1.49E+05	1.55E+05
344.243038	11.67	NH4+	C18H30O5	-1.9	4	0.28	1.67	-1.11	7.18E+06	1.09E+07	5.73E+05	5.87E+05		2.10E+06
348.185862	9.88	NH4+	C12H26O10	-3.2	0	0.83	2.17	-0.50	7.03E+04	1.40E+06	1.64E+05	8.05E+04		1.56E+06
348.188438	10.97	NH4+	C12H26O10	4.2	0	0.83	2.17	-0.50				2.25E+04	2.01E+06	3.42E+04
348.191094	10.96	NH4+	C12H26O10	11.8	0	0.83	2.17	-0.50	1.64E+06	4.06E+04	1.48E+06	4.46E+05	3.75E+05	6.15E+04
351.003004	1.30	Na+	Na4C8H12O8	2.7	4	1.00	1.50	0.50	1.96E+06		9.41E+04			
353.341467	12.57	H+	C23H44O2	-1.4	2	0.09	1.91	-1.74	1.04E+06	1.44E+06	1.22E+06	4.88E+04	1.06E+06	1.52E+06
359.206182	9.43	Na+	C16H32O7	4.5	1	0.44	2.00	-1.13		2.13E+06				
359.315694	12.42	H+	C21H42O4	-1.2	1	0.19	2.00	-1.62	9.06E+04	1.30E+05	7.04E+05	5.63E+05	1.07E+04	7.90E+05
360.214483	11.04	NH4+	C21H26O4	-8.3	9	0.19	1.24	-0.86	3.69E+05	7.93E+05	1.29E+05	5.20E+05	1.13E+05	4.93E+05
362.195166	11.45	NH4+	C20H24O5	-4.4	9	0.25	1.20	-0.70				3.98E+06		3.90E+06
362.197188	11.43	NH4+	C20H24O5	1.2	9	0.25	1.20	-0.70	3.54E+06	2.77E+06	3.88E+06			
362.217979	11.44	NH4+	C17H28O7	0.3	4	0.41	1.65	-0.82					3.90E+06	3.21E+05
365.223083	11.30	ACN-NH4+	C21H22O2	0.5	11	0.09	1.22	-1.39	3.14E+05			1.97E+06	1.72E+05	
365.224978	9.30	ACN-NH4+	C21H22O2	5.7	11	0.10	1.05	-0.86			1.99E+06			2.13E+06
375.213618	10.24	Na+	C20H32O5	-3.0	5	0.25	1.60	-1.10			6.74E+04	1.25E+06		9.96E+05
376.213243	10.81	NH4+	C21H26O5	2.3	9	0.24	1.24	-0.76	1.64E+06			1.95E+06	1.86E+06	1.47E+06

376.342730	12.98	NH4+	C21H42O4	0.1	1	0.19	2.00	-1.62	1.08E+05	6.32E+05	9.56E+05	6.08E+04		7.34E+05
377.226135	9.15	ACN-NH4+	C15H26O7	-7.0	3	0.47	1.73	-0.80		2.52E+06		5.10E+05	3.48E+06	2.52E+06
378.211834	10.92	NH4+	C17H28O8	-2.5	4	0.47	1.65	-0.71	1.01E+07	8.61E+06				
378.214662	11.75	NH4+	C17H28O8	4.9	4	0.47	1.65	-0.71				8.06E+05	3.68E+05	1.08E+07
380.190095	9.02	NH4+	C16H26O9	-5.1	4	0.56	1.63	-0.50	2.77E+06				3.64E+06	
381.114655	7.95	ACN-NH4+	C11H14O11	0.3	5	1.00	1.27	0.73						2.66E+06
383.215136	14.11	ACN-NH4+	C17H24O6	-8.0	6	0.35	1.41	-0.71	1.69E+06		1.23E+06	2.25E+05	5.61E+05	4.86E+04
384.076813	6.46	NH4+	C12H14O13	-2.6	6	1.08	1.17	1.00						2.59E+06
388.253436	8.40	NH4+	C16H34O9	-3.1	0	0.56	2.13	-1.00		1.59E+06		5.88E+05	3.84E+05	5.08E+05
388.269326	11.85	NH4+	C20H34O6	-1.5	4	0.30	1.70	-1.10	7.82E+05	1.44E+06		9.56E+04	1.12E+05	1.64E+05
389.213417	10.25	ACN-NH4+	C12H26O10	-0.3	0	0.83	2.17	-0.50		1.57E+06				1.45E+06
389.215214	14.01	ACN-NH4+	C12H26O10	4.4	0	0.83	2.17	-0.50	1.58E+04		2.22E+04	1.52E+06	1.52E+06	
391.284773	13.51	H+	C24H38O4	-0.2	6	0.17	1.58	-1.25	8.33E+06	8.93E+06	9.13E+04	5.63E+06		8.27E+05
392.211140	10.26	NH4+	C14H30O11	-5.2	0	0.79	2.14	-0.57			2.68E+06			
392.228462	10.30	NH4+	C18H30O8	0.1	4	0.44	1.67	-0.78	1.42E+05	1.62E+05	1.16E+05	4.36E+06		2.41E+06
398.093292	8.30	NH4+	C13H16O13	-0.4	6	1.00	1.23	0.77						2.60E+06
399.250190	11.08	Na+	C23H36O4	-2.3	6	0.17	1.57	-1.22	1.15E+06	9.51E+04	1.17E+05	1.13E+06	1.70E+06	1.17E+05
403.220256	11.39	ACN-NH4+	C20H24O5	-7.5	9	0.25	1.20	-0.70	1.79E+06	7.66E+04				1.77E+06
403.227826	11.44	ACN-NH4+	C20H24O5	-7.5	9	0.25	1.20	-0.70		1.67E+06			1.48E+06	
403.248302	11.40	Na+	C22H36O5	5.6	5	0.23	1.64	-1.18	5.09E+04		1.97E+06		5.37E+04	4.92E+04
408.223877	10.39	NH4+	C18H30O9	1.3	4	0.50	1.67	-0.67	1.34E+05	1.24E+06	1.16E+05	1.72E+06	1.33E+05	1.38E+05
417.240352	10.10	Na+	C26H34O3	-0.5	10	0.12	1.31	-1.08		4.55E+05	2.11E+05	3.27E+06	1.89E+06	
419.245759	11.86	H+	C24H34O6	5.7	8	0.25	1.42	-0.92	1.99E+06	1.43E+05	6.85E+04	4.59E+05	9.99E+05	
419.315815	13.34	Na+	C24H44O4	5.0	3	0.17	1.83	-1.50	5.99E+04	2.09E+06	5.72E+04		1.94E+05	
421.215731	9.05	Na+	C28H30O2	3.3	14	0.07	1.07	-0.93	4.37E+06	3.55E+06	4.90E+06	3.85E+06	4.38E+06	4.07E+05

429.242957	12.67	Na+	C27H34O3	5.6	11	0.11	1.26	-1.04	4.19E+05	4.74E+03		8.75E+05	7.30E+05	
436.234051	11.62	NH4+	C23H30O7	1.2	9	0.30	1.30	-0.70	2.90E+06	1.02E+05	1.21E+05		2.17E+06	
436.236710	11.31	NH4+	C16H34O12	-6.2	0	0.75	2.13	-0.63		2.23E+04		2.43E+06		3.20E+06
437.434324	13.03	H+	C29H56O2	-3.5	2	0.07	1.93	-1.79	8.43E+04	4.38E+04	8.41E+05	6.86E+04	6.04E+05	1.03E+06
439.119812	8.30	ACN-NH4+	C13H16O13	-0.5	6	1.00	1.23	0.77						2.29E+06
439.259207	11.50	Na+	C29H36O2	-4.8	12	0.07	1.24	-1.10	1.26E+06		2.09E+06		3.02E+05	1.24E+06
442.082458	6.90	NH4+	C14H16O15	-1.9	7	1.07	1.14	1.00						3.24E+06
449.337527	12.15	Na+	C29H46O2	-4.5	7	0.07	1.59	-1.45	1.14E+06	1.20E+06	2.75E+04	1.38E+05		
450.248566	11.07	NH4+	C24H32O7	-1.4	9	0.29	1.33	-0.75	2.07E+05			1.03E+06	7.91E+05	
452.228760	11.52	NH4+	C23H30O8	0.7	9	0.35	1.30	-0.61				2.28E+06	6.39E+05	5.45E+05
452.249190	11.58	NH4+	C20H34O10	-0.8	4	0.50	1.70	-0.70	2.22E+06					3.30E+05
454.459488	13.08	NH4+	C29H56O2	-6.4	2	0.07	1.93	-1.79	1.40E+06	1.94E+06	7.73E+05	2.10E+05	9.09E+05	1.20E+06
458.275669	12.75	NH4+	C23H36O8	0.6	6	0.35	1.57	-0.87	8.26E+05	4.87E+05	5.05E+05	2.76E+05	4.53E+05	4.76E+05
461.266269	10.21	Na+	C28H38O4	-1.1	10	0.14	1.36	-1.07		9.78E+05	6.56E+04	1.86E+06	8.35E+05	
466.222223	11.51	NH4+	C27H28O6	-1.6	14	0.22	1.04	-0.59	1.81E+06	1.56E+06	1.80E+06	1.62E+05		1.73E+06
473.265171	14.54	Na+	C29H38O4	-3.4	11	0.14	1.31	-1.03	7.10E+05	1.86E+05	5.41E+05			8.54E+05
475.277509	13.44	H+	C20H42O12	4.3	0	0.60	2.10	-0.90	5.67E+05		3.99E+05	1.45E+06		
479.277702	13.36	ACN-NH4+	C23H32O7	4.1	8	0.30	1.39	-0.78				2.29E+05	7.55E+04	1.75E+06
485.357428	14.85	ACN-NH4+	C24H42O6	-3.4	4	0.25	1.75	-1.25	6.82E+05		4.19E+03	6.35E+05	9.02E+05	1.04E+06
486.307671	13.52	NH4+	C25H40O8	2.0	6	0.32	1.60	-0.96	7.39E+05	4.32E+04	6.12E+05	4.56E+05	2.12E+05	1.90E+05
513.286219	10.09	Na+	C28H42O7	6.6	8	0.25	1.50	-1.00		4.19E+05	3.06E+04	2.05E+06	1.48E+06	

The start time (or beginning of data acquisition) for each experiment is listed in each figure caption. After starting data acquisition, the chamber was filled with N_2 and O_2, the automatic pressure regulation system was turned on, analytical instruments were connected to the chamber, and "blank" data was recorded. This startup process a few hours to complete; data from this period is not shown in the figures.

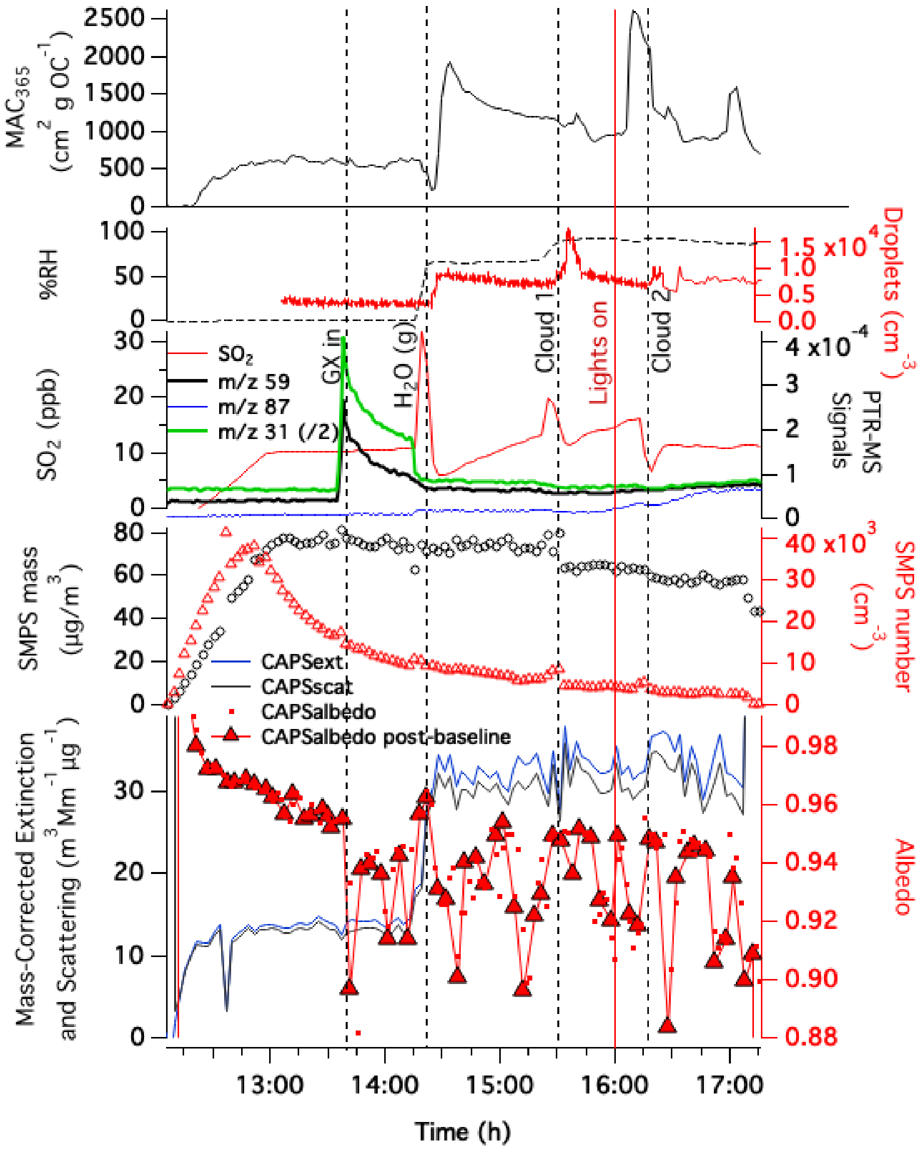

Figure S1: Experiment 6 with pH 5.5 Na_2SO_3 aerosol, glyoxal (g), 1 dark and 1 light cloud without HOOH, 10:11 start time. Top panel: mass absorption coefficients measured by PILS – waveguide - TOC at 365 nm (black line). 2nd panel: chamber RH and droplet spectrometer counts, color-coded to axes. 3rd panel: water- and dilution-corrected PTR-MS data from chamber (glyoxal signals *m/z* 31 and 59, green and black lines, respectively; *m/z* 87, blue line), and SO_2 concentrations in ppb from dedicated sensor (red line). 4th panel: SMPS total mass (assuming density = 1 g/cm^3) and counts, color-coded to axes. Bottom: CAPS-ssa data at 450 nm (mass-corrected extinction, blue line; mass-corrected scattering, black line; 2-min averaged single-scattering albedo, red dots, and albedo measured immediately after instrument baselines, red triangles). Glyoxal pulse addition, water vapor addition, two cloud events, and the start of chamber illumination by solar simulator lights are labeled. Glyoxal was also added continuously after 15:20.

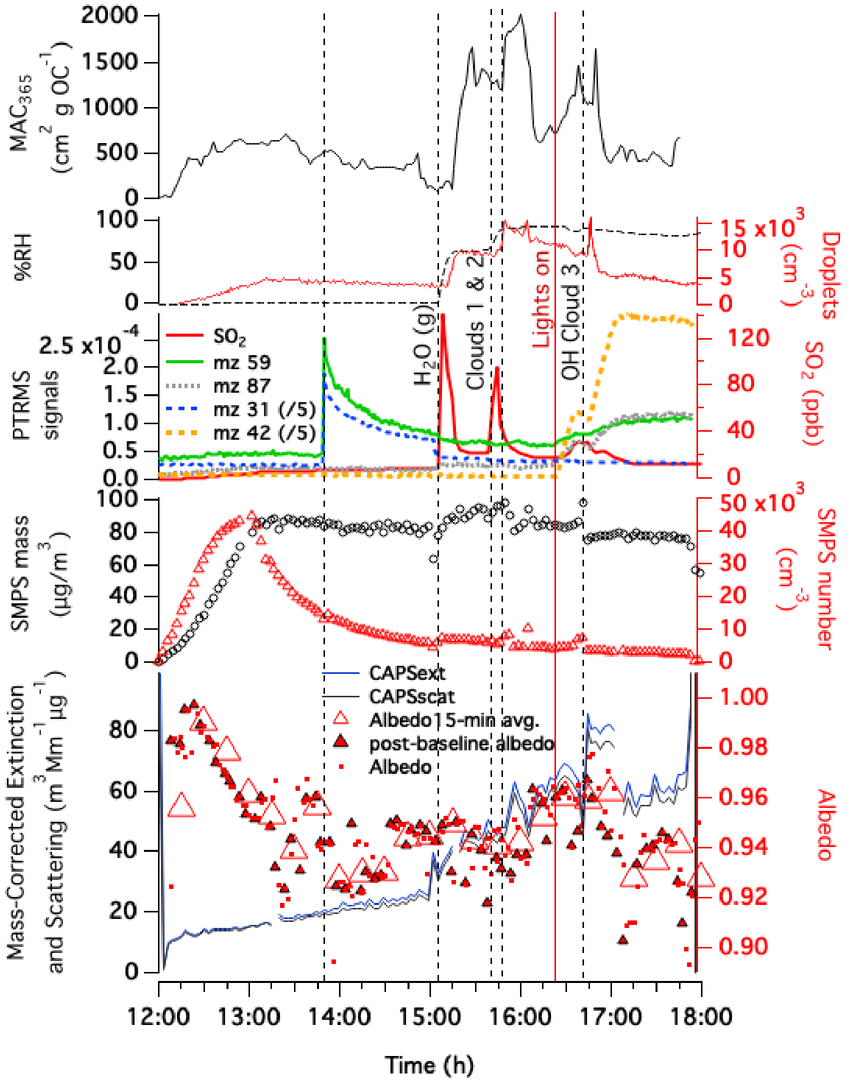

Figure S2: Experiment 5 with pH 5.5 Na_2SO_3 aerosol, glyoxal (g), 2 dark and 1 photooxidative cloud, 10:15 start time. Top panel: mass absorption coefficients measured by PILS – waveguide - TOC at 365 nm (black line). 2nd panel: chamber RH and droplet spectrometer counts, color-coded to axes. 3rd panel: water- and dilution-corrected PTR-MS data from chamber (glyoxal signals *m/z* 31 and 59, solid green and dashed blue lines, respectively; *m/z* 42, dotted orange line; *m/z* 87, dotted gray line), and SO_2 concentrations in ppb from dedicated sensor (red line). 4th panel: SMPS total mass (assuming density = 1 g/cm^3) and counts, color-coded to axes. Bottom: CAPS-ssa data at 450 nm (mass-corrected extinction, blue line; mass-corrected scattering, black line; 2-min averaged single-scattering albedo, red dots; 15-min averaged albedo, large open red triangles; and albedo measured immediately after instrument baselines, filled red triangles). Glyoxal pulse addition, water vapor addition, three cloud events, and the start of chamber illumination by solar simulator lights are labeled.

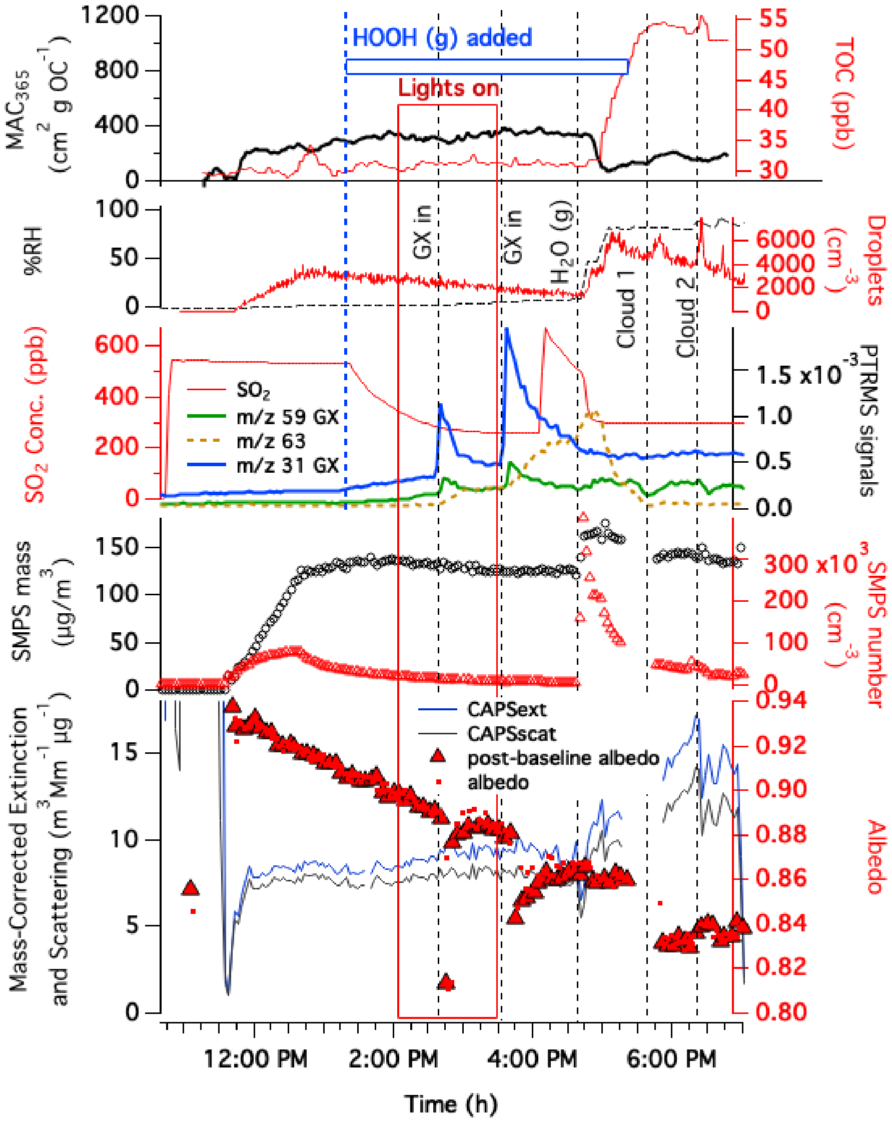

Figure S3: Experiment 2 with SO_2(g) + Na_2SO_4 seed aerosol + HOOH(g) + glyoxal(g) + lights + dark clouds, 8:20 start time. Top panel: mass absorption coefficients measured by PILS – waveguide - TOC at 365 nm (black line), and TOC data (red line). 2nd panel: chamber RH and droplet spectrometer counts, color-coded to axes. 3rd panel: water- and dilution-corrected PTR-MS data from chamber (glyoxal signals *m/z* 31 and 59, blue and green lines, respectively; *m/z* 63, red line), and SO_2 concentrations in ppb from dedicated sensor (black line). 4th panel: SMPS total mass (assuming density = 1 g/cm^3) and counts, color-coded to axes. Bottom: CAPS-ssa data at 450 nm (mass-corrected extinction, blue line; mass-corrected scattering, black line; 2-min averaged single-scattering albedo, red dots, and albedo measured immediately after instrument baselines, red triangles). Additions of HOOH, glyoxal, water vapor, two cloud events, and chamber illumination period by solar simulator lights, are labeled. New particle nucleation of non-absorbing sulfate aerosol occurred upon chamber humidification to 25% RH.

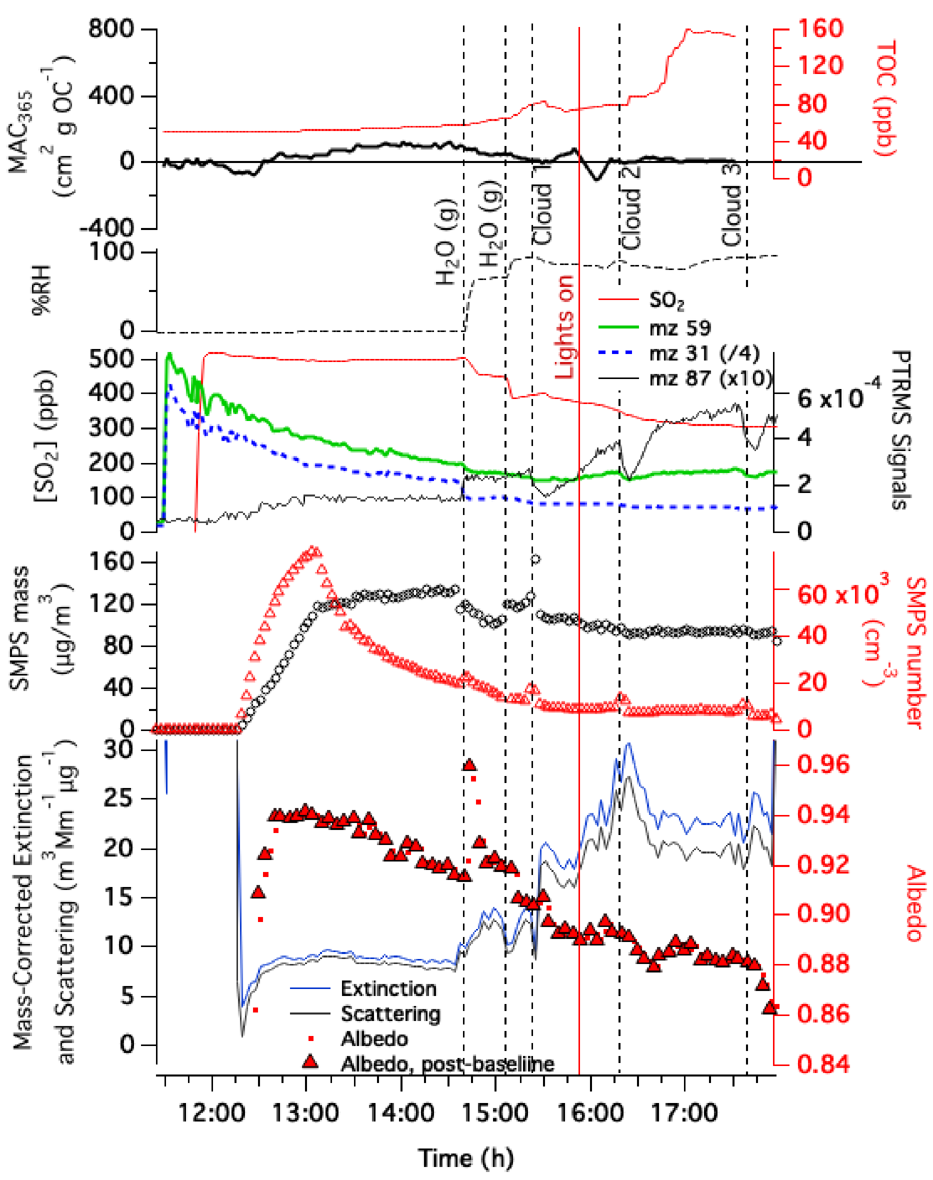

Figure S4: Experiment 3 with glyoxal(g) + SO_2(g) + Na_2SO_4 seeds + 1 dark and 1 photolytic cloud, 8:18 start time. No HOOH(g) added. Top panel: mass absorption coefficients measured by PILS – waveguide - TOC at 365 nm (black line), and TOC data (red line). 2nd panel: chamber relative humidity. 3rd panel: water- and dilution-corrected PTR-MS data from chamber (*m/z* 87, black line, data multiplied by 10; glyoxal signals *m/z* 31 (divided by 4) and 59, blue dashes and green line, respectively), and SO_2 concentrations in ppb from dedicated sensor (red line). 4th panel: SMPS total mass (assuming density = 1 g/cm^3) and counts, color-coded to axes. Bottom: CAPS-ssa data at 450 nm (mass-corrected extinction, blue line; mass-corrected scattering, black line; 2-min averaged single-scattering albedo, red dots, and albedo measured immediately after instrument baselines, red triangles). Water vapor addition, three cloud events, and start of chamber illumination by solar simulator lights are labeled.

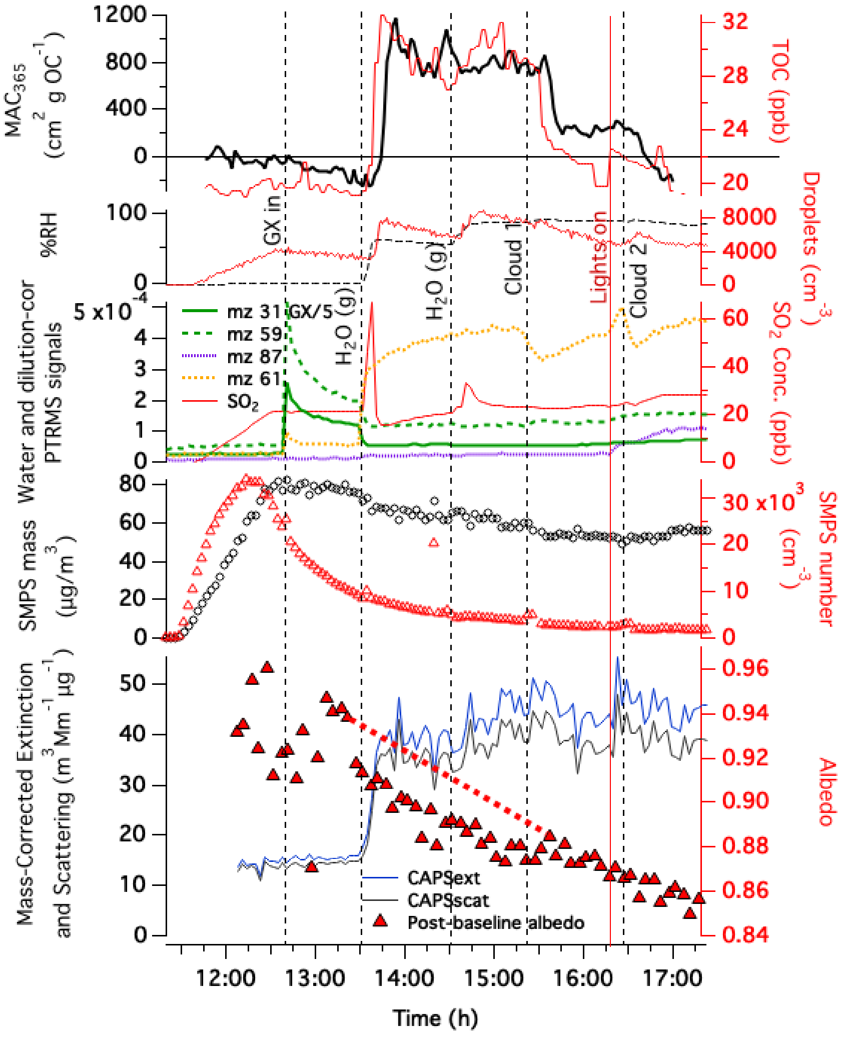

Figure S5: Experiment 7 with Na_2SO_3 aerosol at pH 5.5 in N_2-filled CESAM chamber, glyoxal(g), 1 dark and 1 light cloud without HOOH, 9:26 start time. Top panel: mass absorption coefficients measured by PILS – waveguide - TOC at 365 nm (black line), and TOC data (red line). 2nd panel: chamber RH and droplet spectrometer counts, color-coded to axes. 3rd panel: water- and dilution-corrected PTR-MS data from chamber (*m/z* 42, orange dotted line, data divided by 5; glyoxal signals *m/z* 31 and 59, blue dashes and green line, respectively; *m/z* 61, gray line), and SO_2 concentrations in ppb from dedicated sensor (red line). 4th panel: SMPS total mass (assuming density = 1 g/cm^3) and counts, color-coded to axes. Bottom: CAPS-ssa data at 450 nm (mass-corrected extinction, blue line; mass-corrected scattering, black line; 2-min averaged single-scattering albedo, red dots, and albedo measured immediately after instrument baselines, red triangles). Glyoxal addition, water vapor addition, two cloud events, start of chamber illumination by solar simulator lights, and the onset of aerosol drying on the way to SMPS and CAPS-ssa instruments are labeled.

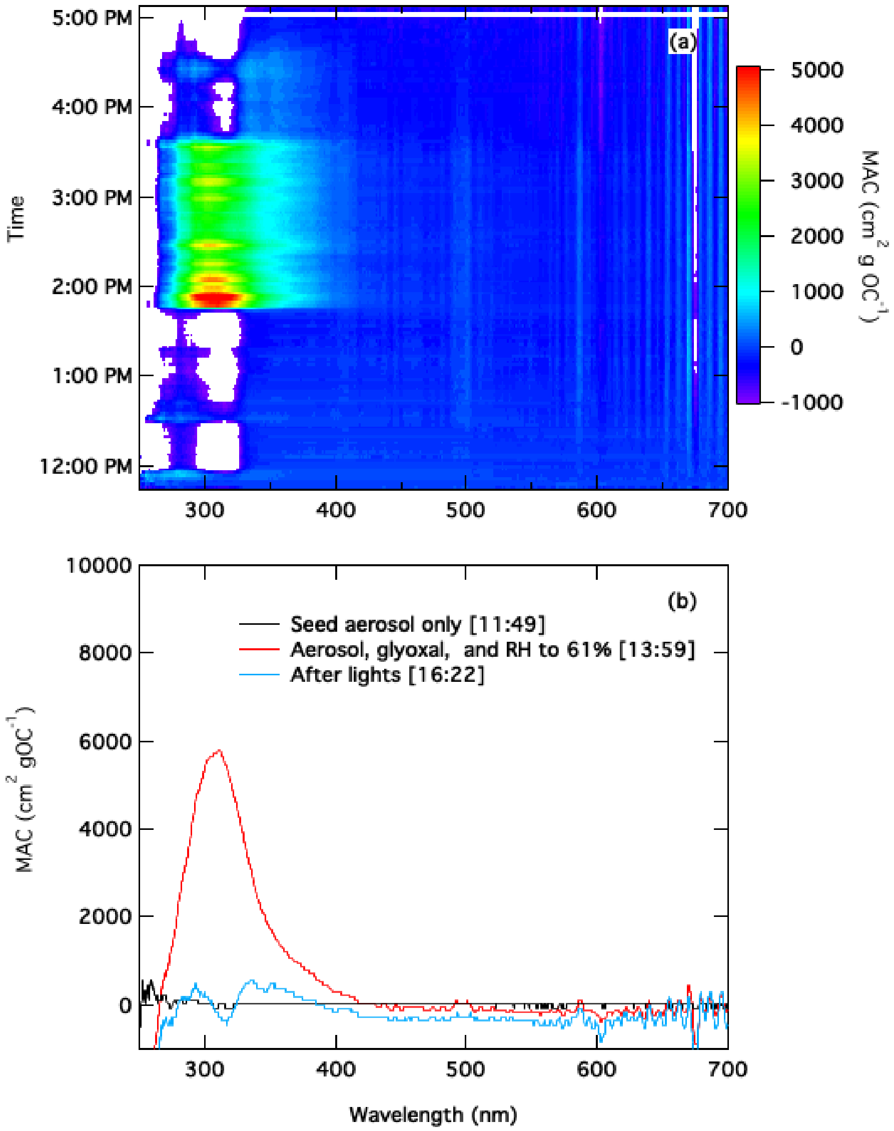

Figure S6: (a) Time-dependent absorbance spectra measured by waveguide spectrometer in PILS outflow during experiment 7 (Na_2SO_3 aerosol at pH 5.5 in N_2-filled CESAM chamber, glyoxal(g), 1 dark and 1 light cloud without HOOH). Large increase in absorbance at 13:40 followed increase in CESAM chamber humidity from < 5% to 61% RH; (b) three representative spectra collected with seed aerosol only (black trace), following the humidification to 61% (red trace) and after a brief cloud formed and chamber lights were turned on (blue trace). Particle losses following a cloud and the general dilution of the chamber left little aerosol mass by the end of the experiment. Absorbance values below zero resulted from small drifts in the performance of the lamp and detector following a reference blank collected on the clean chamber, which result from changes to the temperature of the laboratory. MAC values are therefore minimum. The drift is most visible in Figure S5 MAC (black) trace and is smaller than changes to the absorptivity signal.

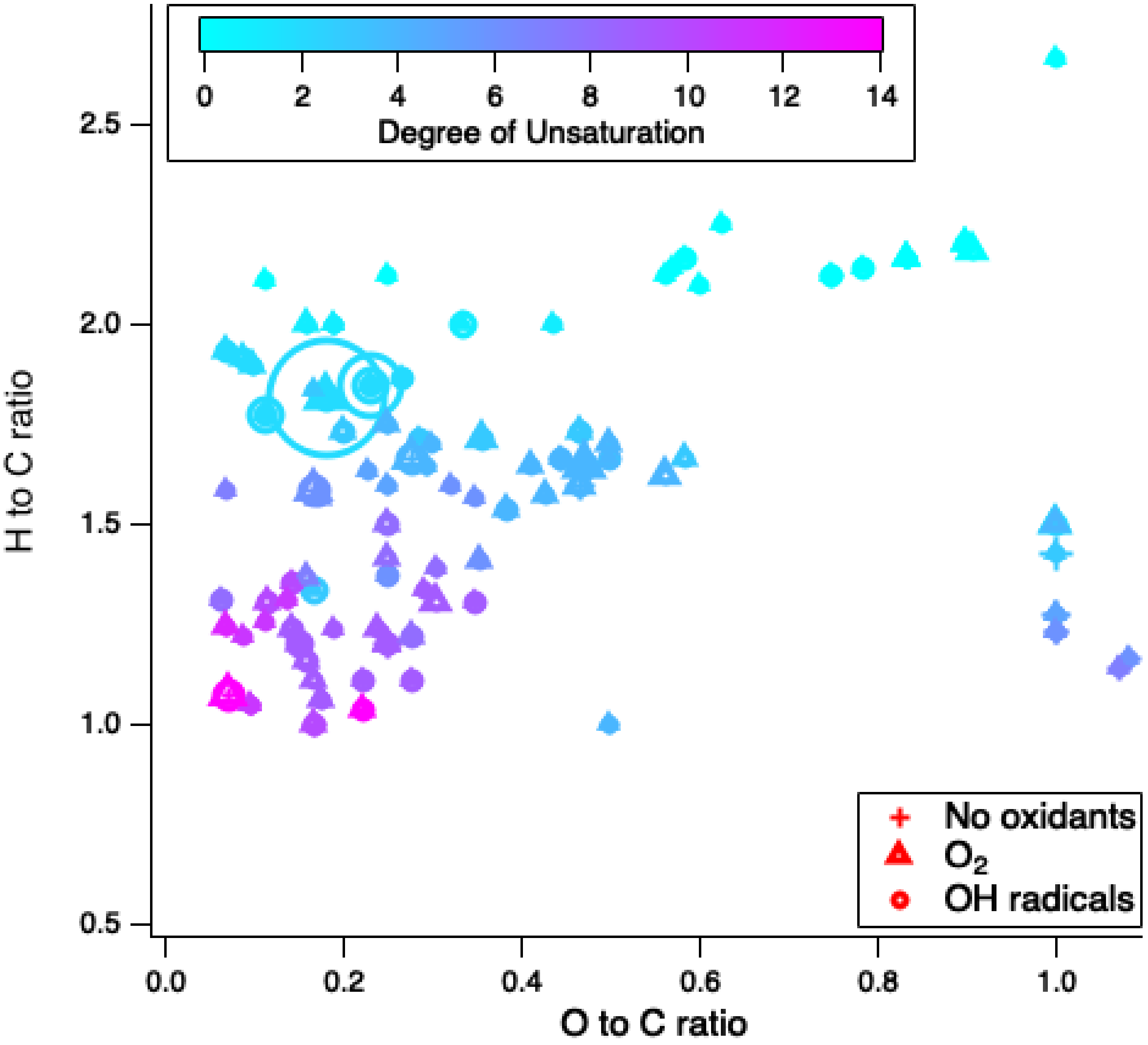

Figure S7: Summary of molecular formula detected by UHPLC/(+)ESI-HR-QTOFMS analysis of extracts of chamber aerosol in experiments 1 and 3-7, graphed in terms of H/C and O/C ratios. Symbols indicate oxidant present: no oxidant (+), and O_2 (triangles), or OH radicals (circles). Colors indicate degrees of unsaturation, and symbol areas are proportional to peak area sums across experiments with the given oxidant. Glyoxal oligomers would appear on the right side of the graph with O/C ratios near 1. Multiple symbols of the same shape and color centered on one location represent isomers with identical mass but distinguished by retention times.
